# Supplementary figures and images for: Global cellular response to chemical perturbation of PLK4 activity and abnormal centrosome number
Source: eLife. 2022 Jun 27;11:e73944. doi: 10.7554/eLife.73944 (PMC9236612; doi:10.7554/eLife.73944)

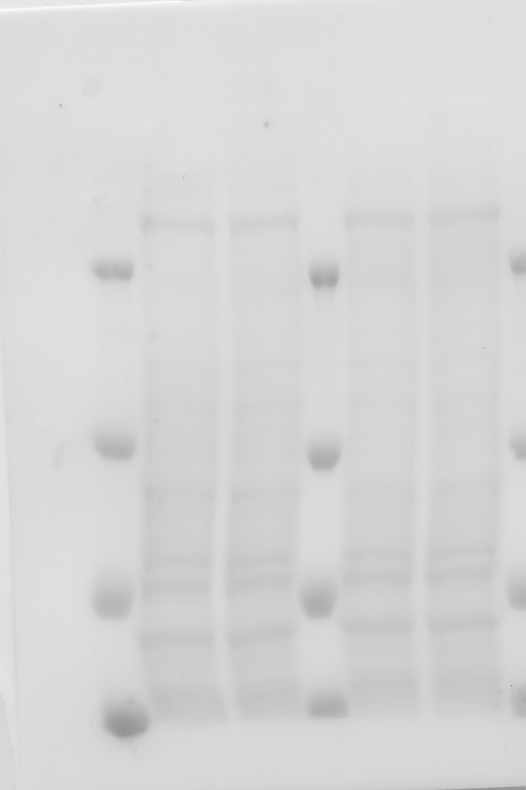

Supplement: Source data 1. [file elife-73944-data1.zip › Western_files/FigureS6_1A/rep3_PCNT_CEP120_ponceau.tif]

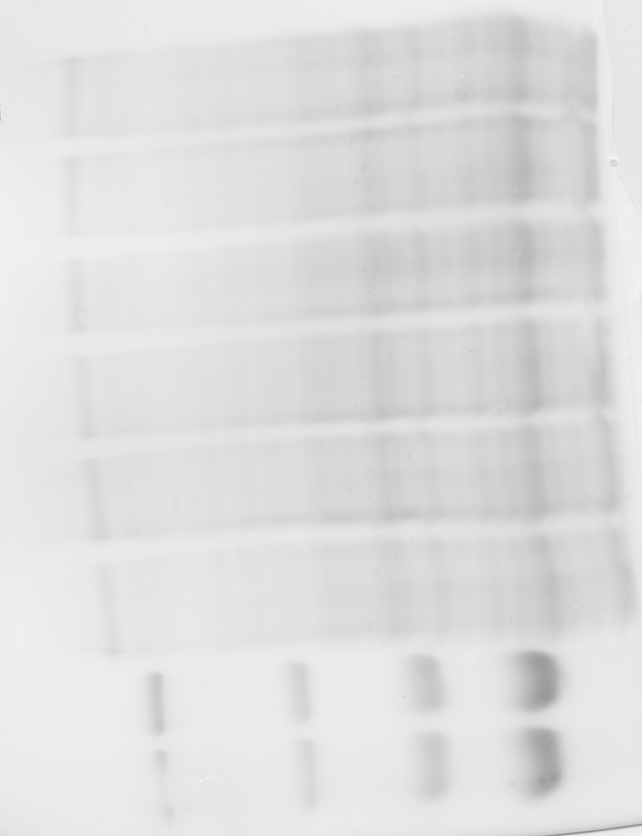

Supplement: Source data 1. [file elife-73944-data1.zip › Western_files/FigureS6_1A/rep1_rep2_CEP120_ponceau.tif]

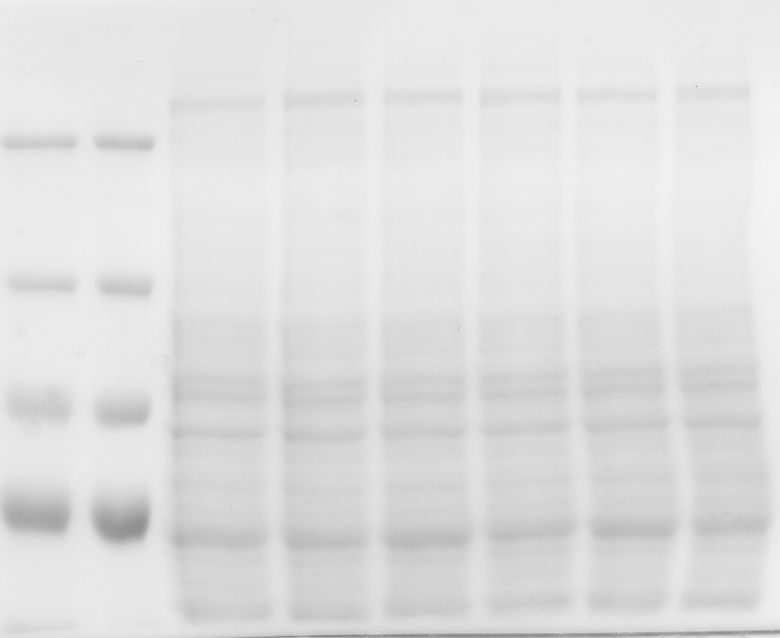

Supplement: Source data 1. [file elife-73944-data1.zip › Western_files/FigureS6_1A/rep1_rep2_PCNT_ponceau.tif]

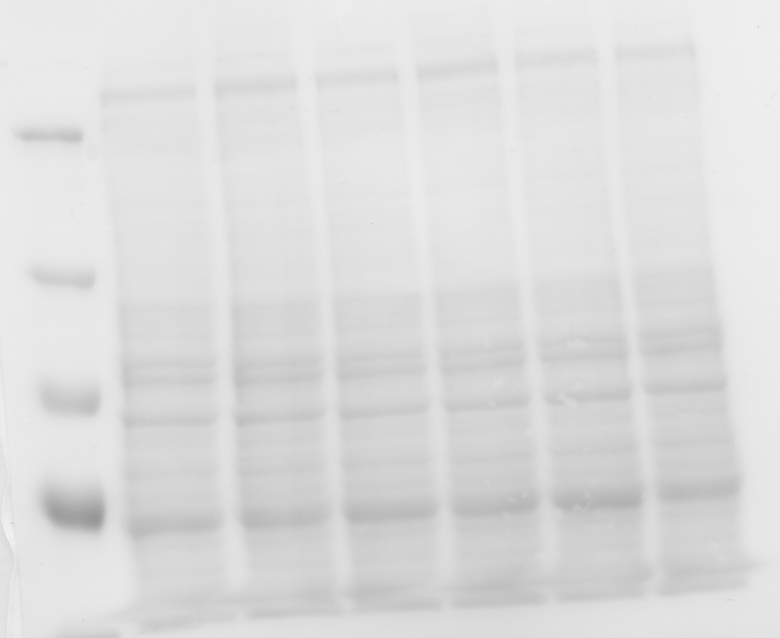

Supplement: Source data 1. [file elife-73944-data1.zip › Western_files/FigureS6_1A/rep1_rep2_rep3_CEP192_ponceau.tif]

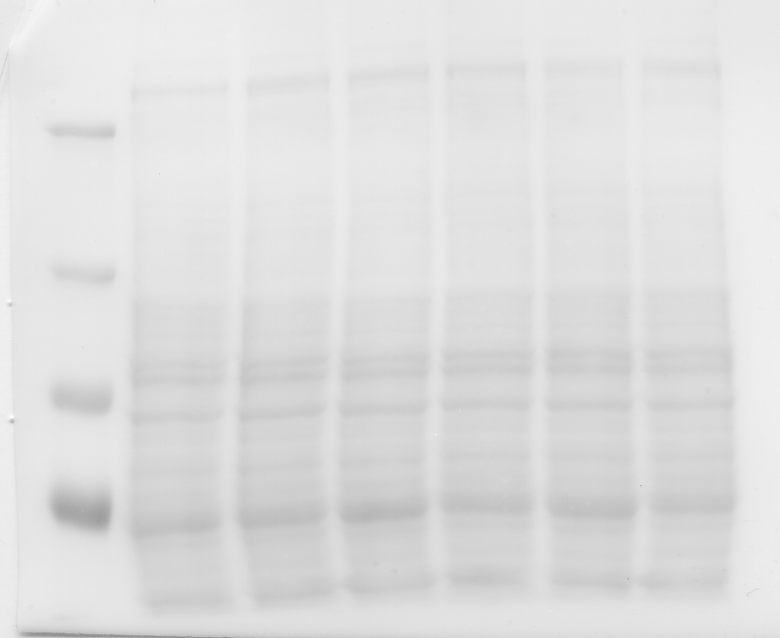

Supplement: Source data 1. [file elife-73944-data1.zip › Western_files/FigureS6_1A/rep1_rep2_rep3_CEP215_ponceau.tif]

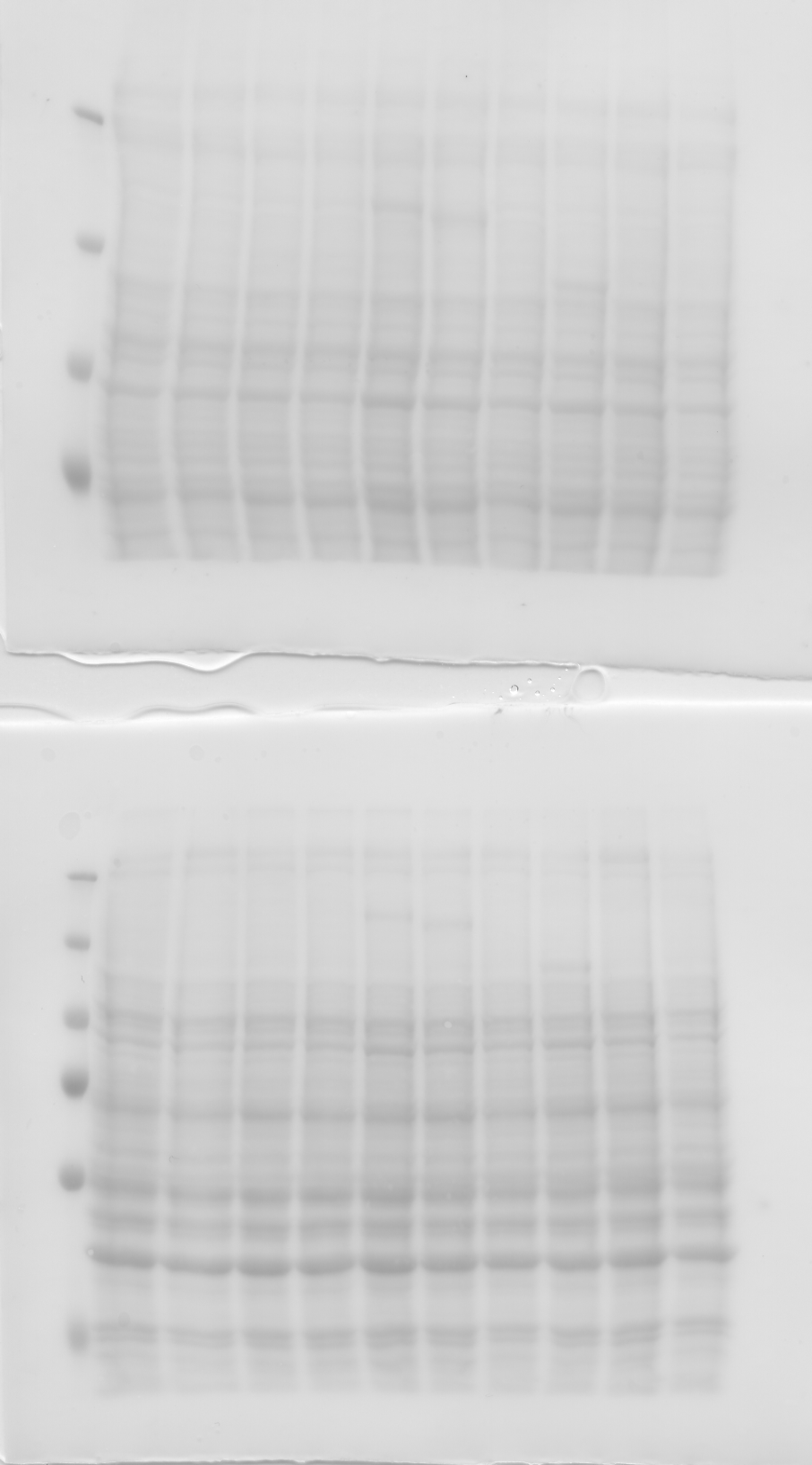

Supplement: Source data 1. [file elife-73944-data1.zip › Western_files/FigureS3_2A/ponceau.tiff]

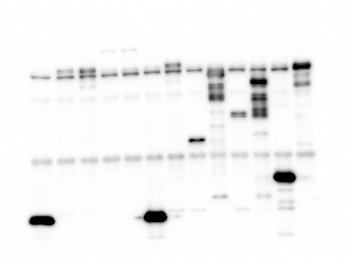

Supplement: Source data 1. [file elife-73944-data1.zip › Western_files/FigureS7_1A/FLAG_IP.tif]

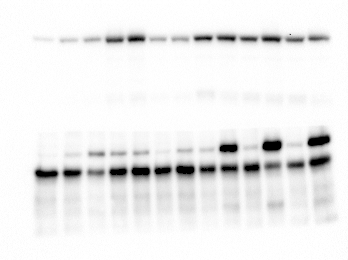

Supplement: Source data 1. [file elife-73944-data1.zip › Western_files/FigureS7_1A/PLK4_Exposure_34s.tif]

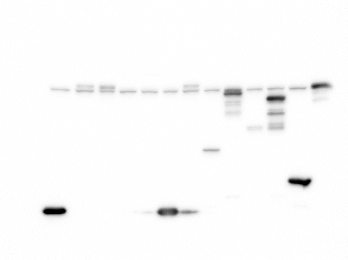

Supplement: Source data 1. [file elife-73944-data1.zip › Western_files/FigureS7_1A/FLAG_input.tif]

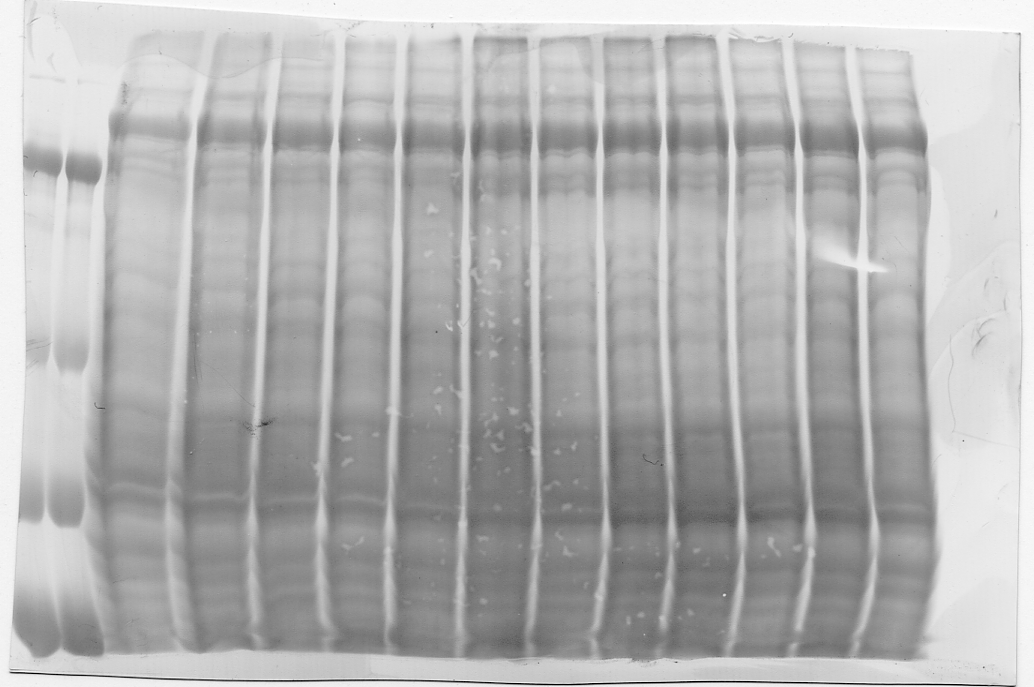

Supplement: Source data 1. [file elife-73944-data1.zip › Western_files/FigureS7_1F/coomassie.tif]

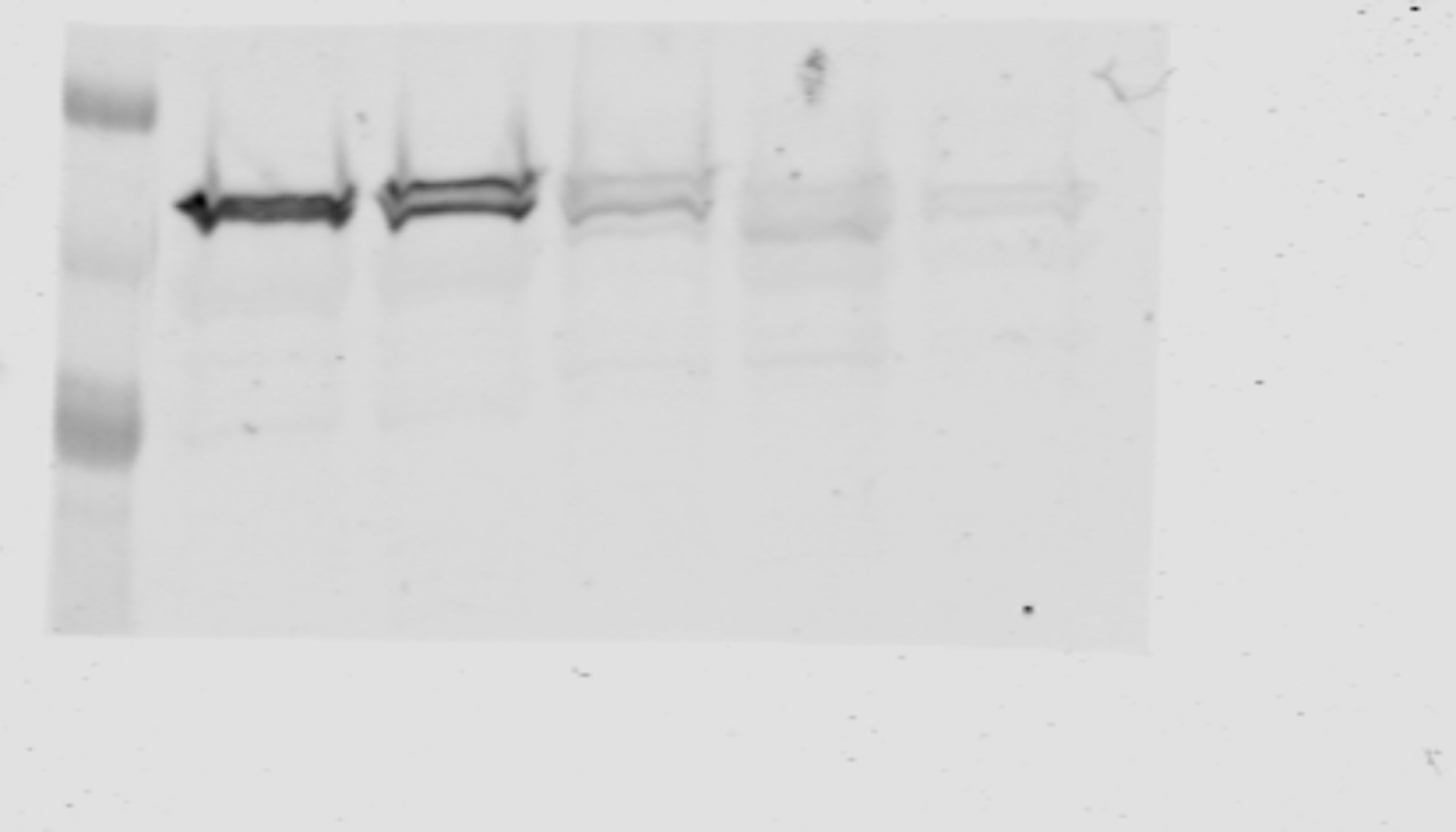

Supplement: Source data 1. [file elife-73944-data1.zip › Western_files/FigureS4_3A/ponceau.tif]

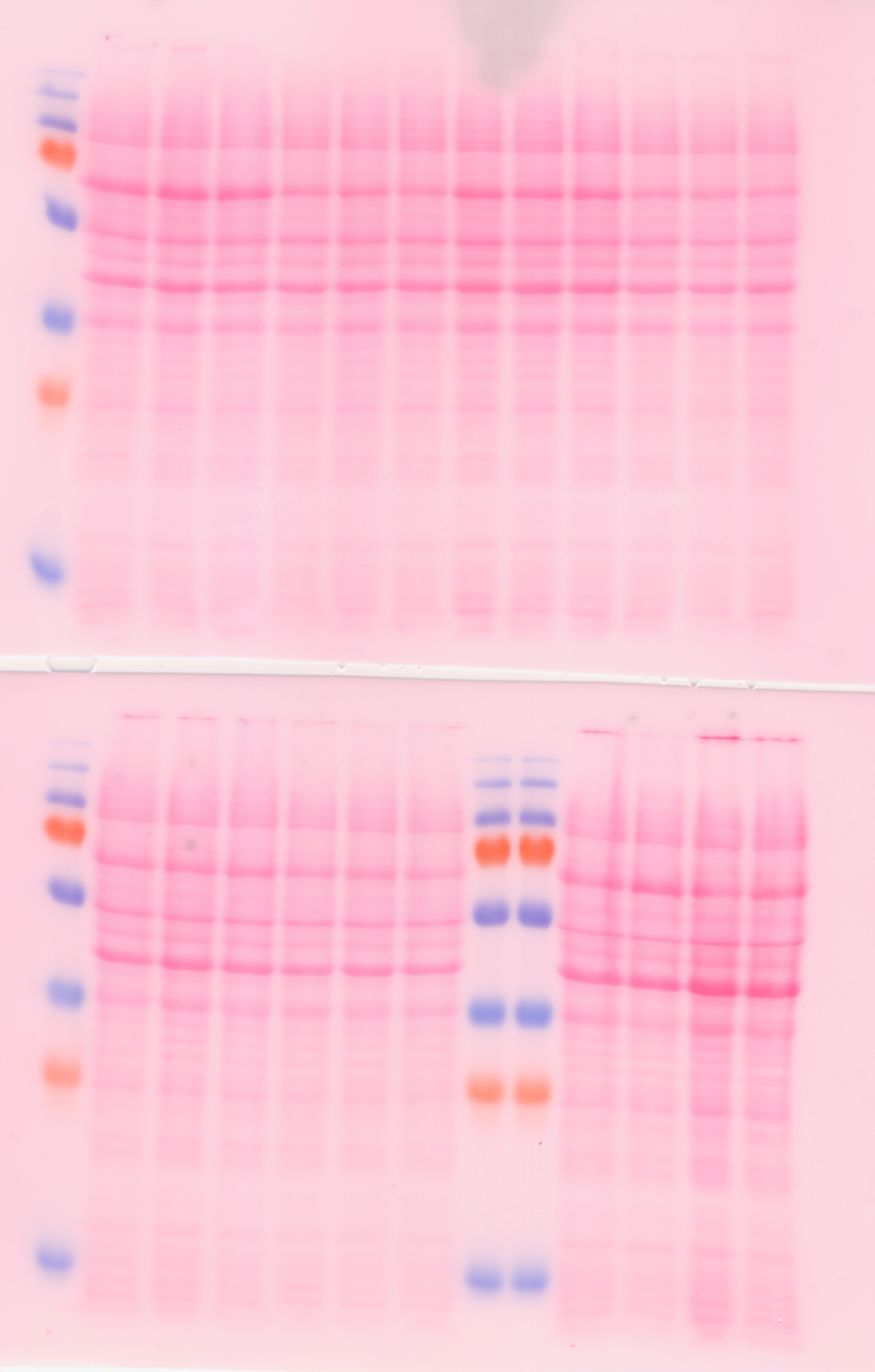

Supplement: Source data 1. [file elife-73944-data1.zip › Western_files/Figure3B/p53_p21_Ponceau.tif]

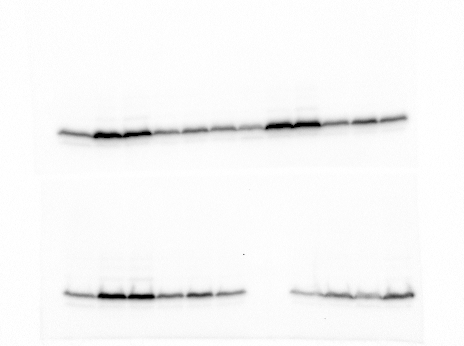

Supplement: Source data 1. [file elife-73944-data1.zip › Western_files/Figure3B/p53_30s.tif]

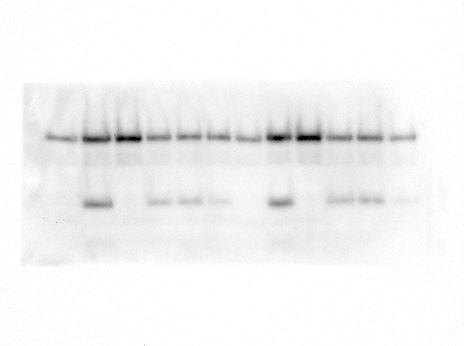

Supplement: Source data 1. [file elife-73944-data1.zip › Western_files/Figure3B/mdm2_110s.tif]

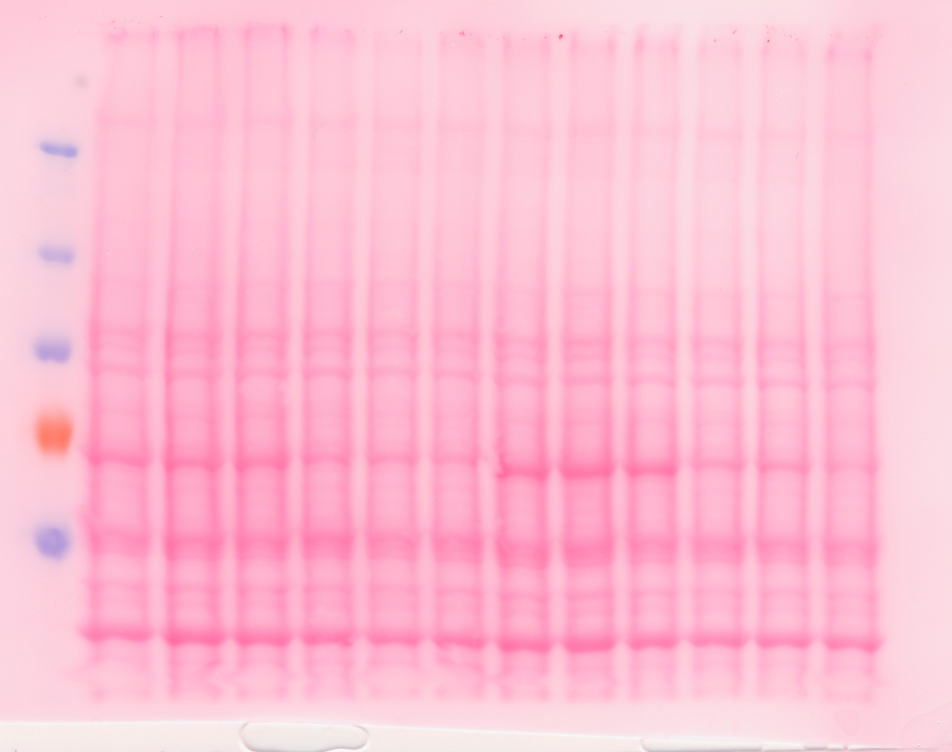

Supplement: Source data 1. [file elife-73944-data1.zip › Western_files/Figure3B/mdm2_ponceau.tif]

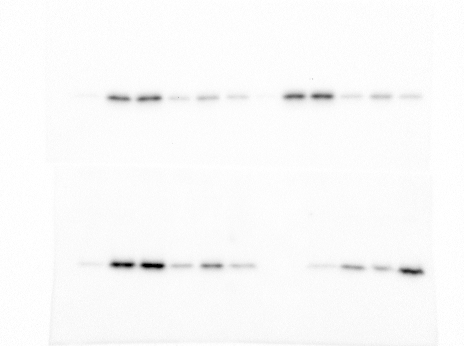

Supplement: Source data 1. [file elife-73944-data1.zip › Western_files/Figure3B/p21_40s.tif]

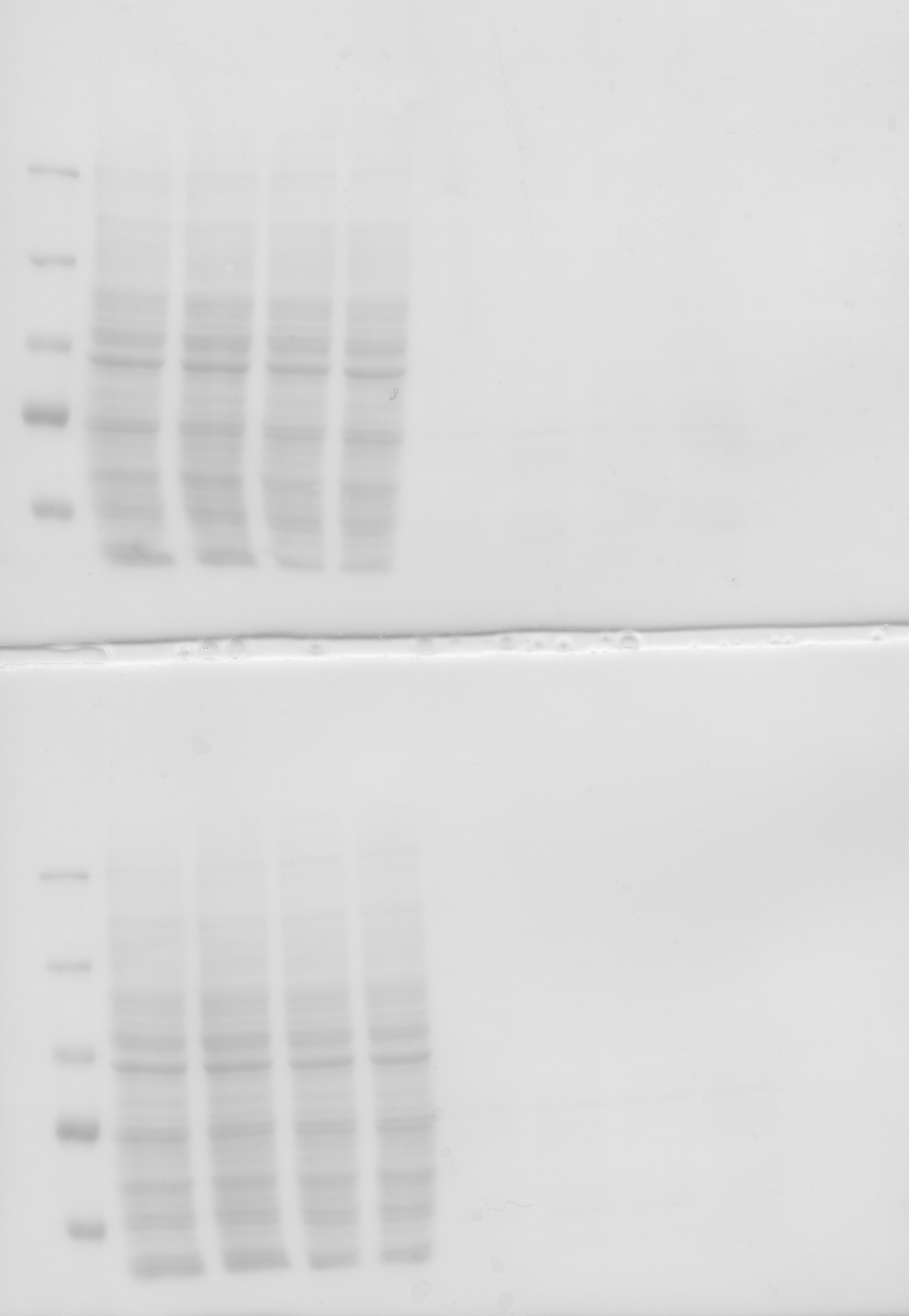

Supplement: Source data 1. [file elife-73944-data1.zip › Western_files/Figure7E/ponceau.tiff]

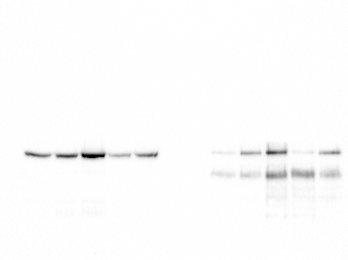

Supplement: Source data 1. [file elife-73944-data1.zip › Western_files/Figure7B/PLK4_Exposure_30.tif]

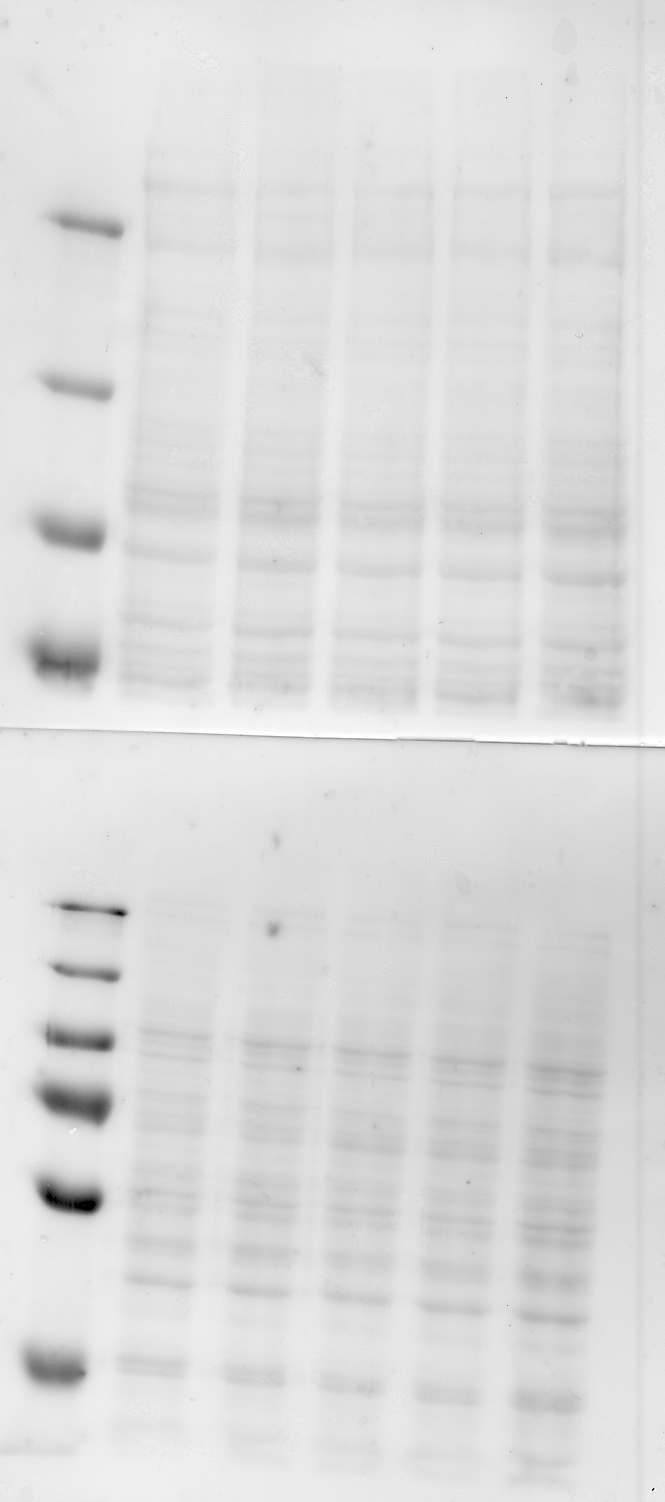

Supplement: Source data 1. [file elife-73944-data1.zip › Western_files/Figure7B/ponceau.TIF]

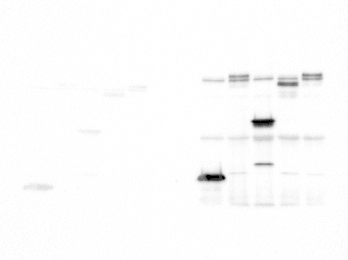

Supplement: Source data 1. [file elife-73944-data1.zip › Western_files/Figure7B/FLAG_Exposure_7.tif]

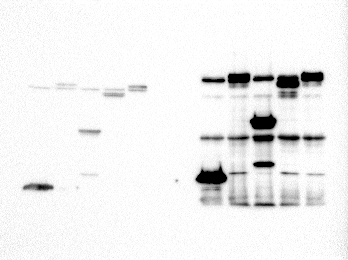

Supplement: Source data 1. [file elife-73944-data1.zip › Western_files/Figure7B/FLAG_Exposure_60.0sec.tif]

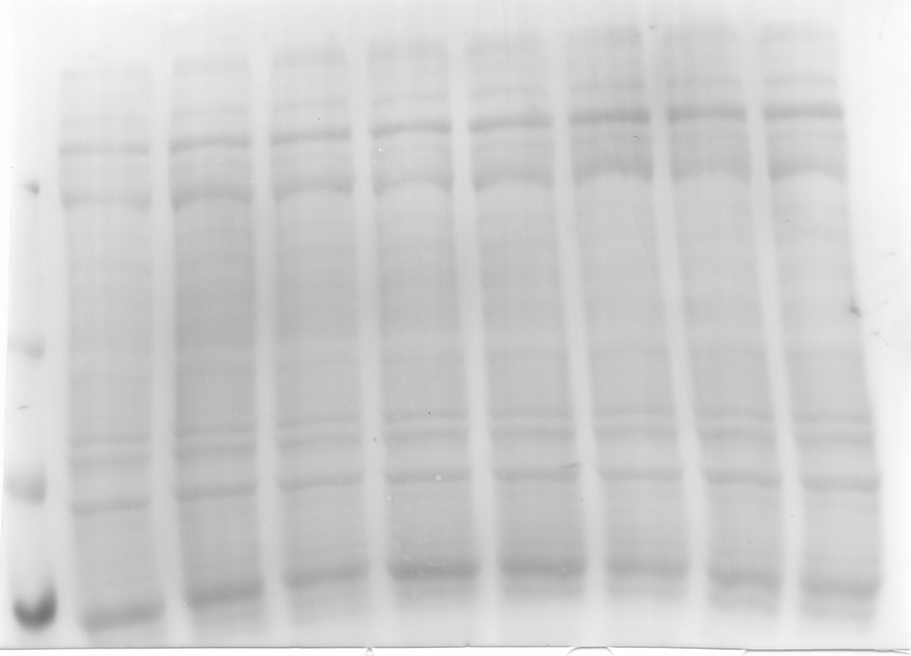

Supplement: Source data 1. [file elife-73944-data1.zip › Western_files/FigureS4_2D/ponceau.tif]

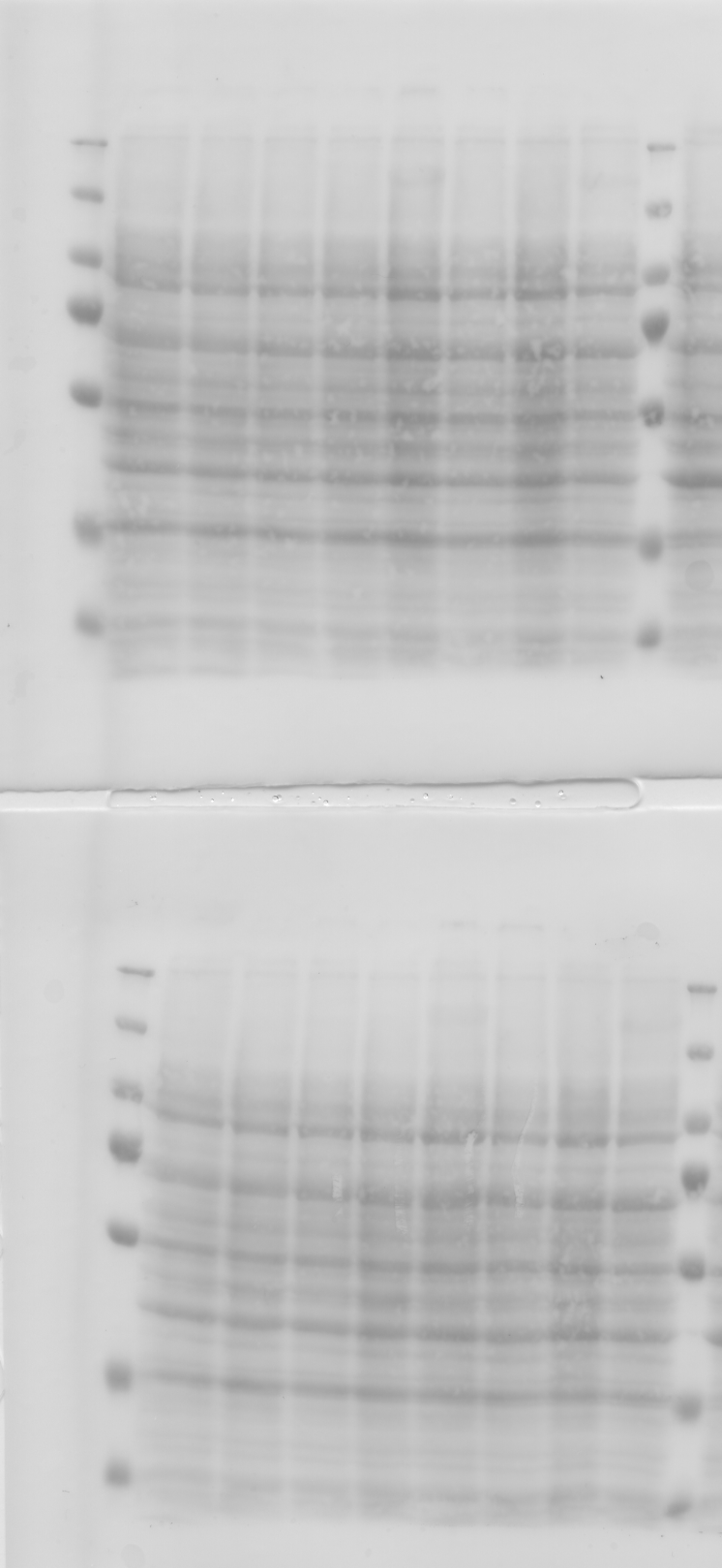

Supplement: Source data 1. [file elife-73944-data1.zip › Western_files/FigureS4_1B/ponceau.tiff]

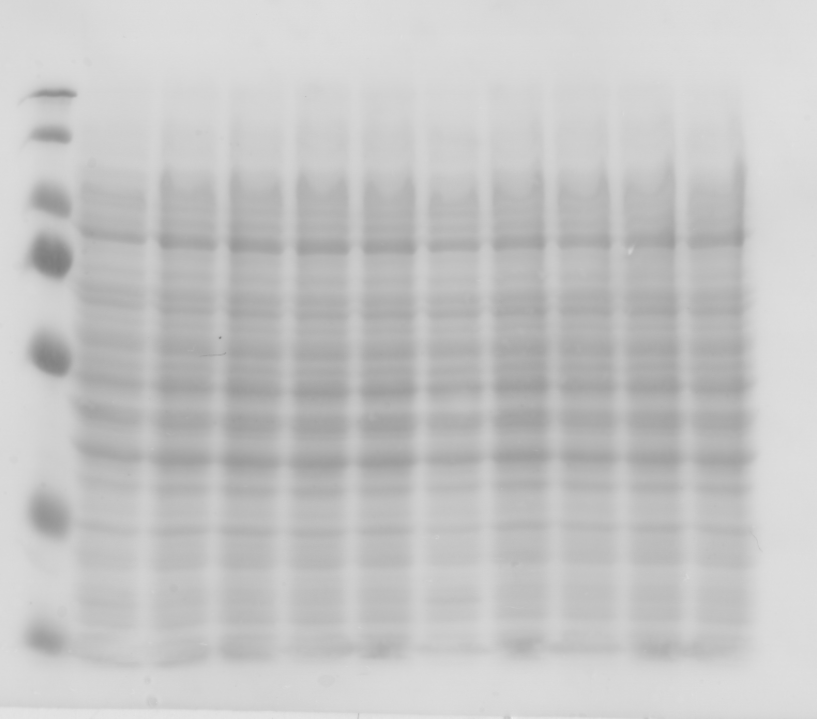

Supplement: Source data 1. [file elife-73944-data1.zip › Western_files/Figure7D/T7_ponceau.tiff]

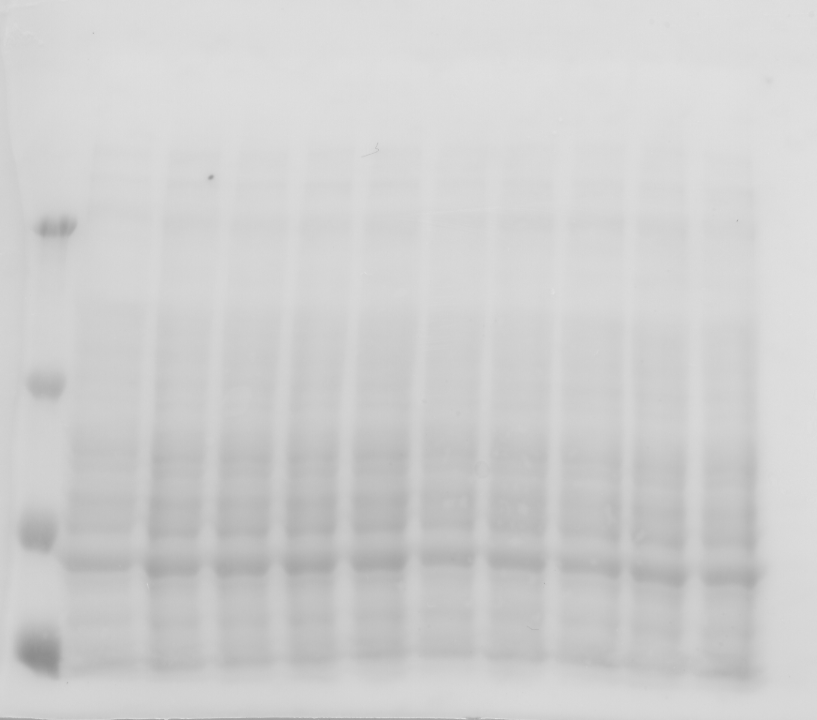

Supplement: Source data 1. [file elife-73944-data1.zip › Western_files/Figure7D/PLK4_ponceau.tiff]

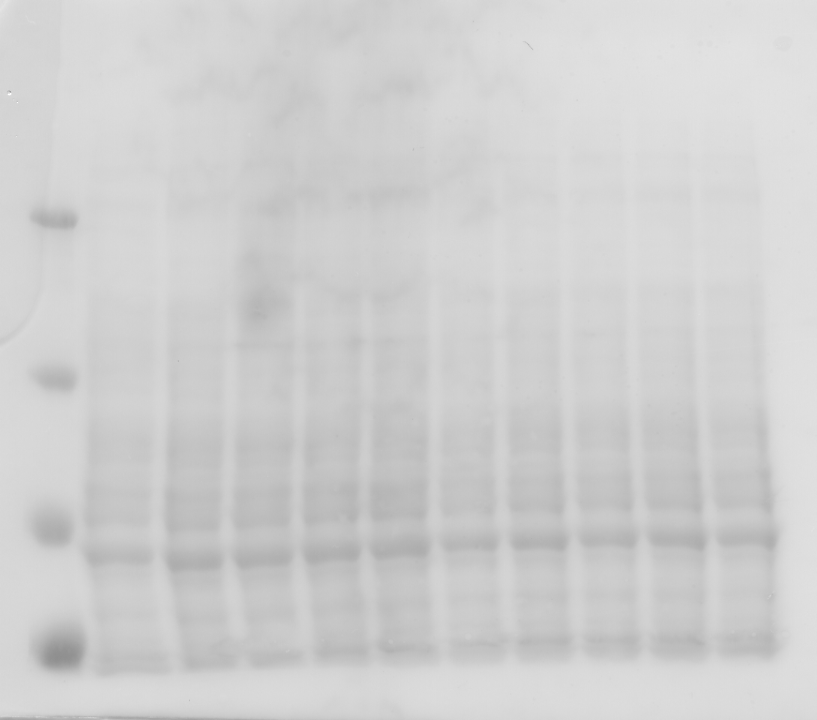

Supplement: Source data 1. [file elife-73944-data1.zip › Western_files/Figure7D/NEDD8_ponceau.tiff]

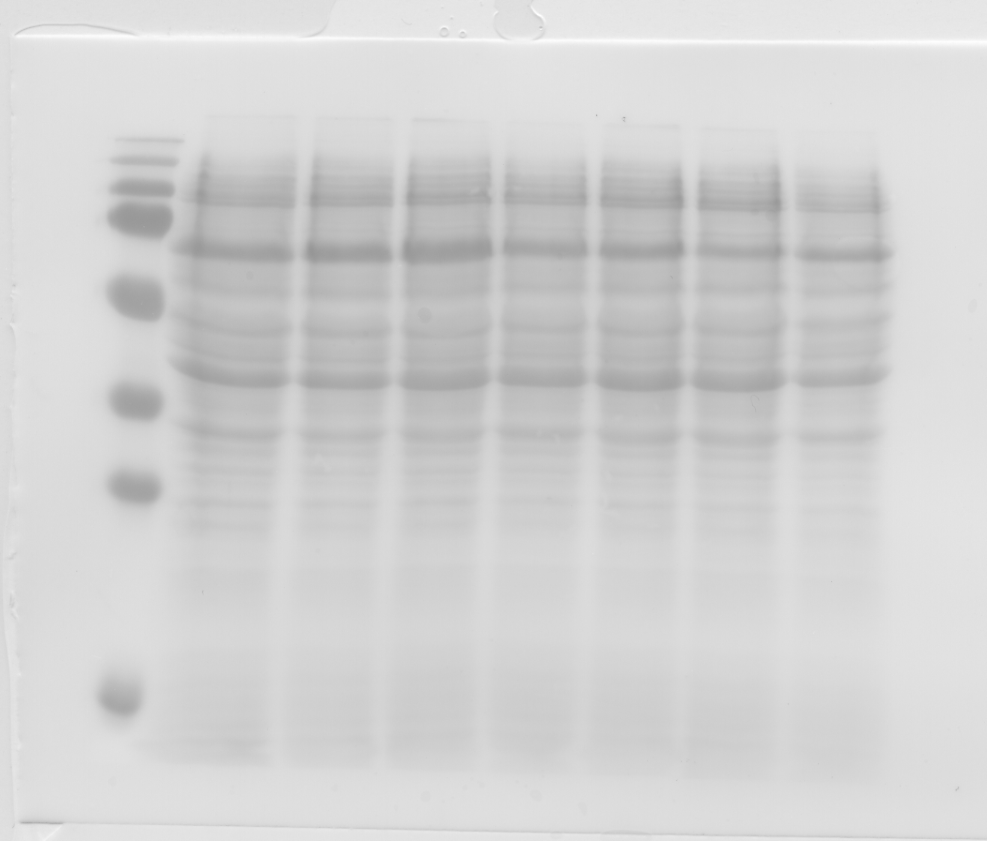

Supplement: Source data 1. [file elife-73944-data1.zip › Western_files/FigureS4_1D/wt_rep3_ponceau.tiff]

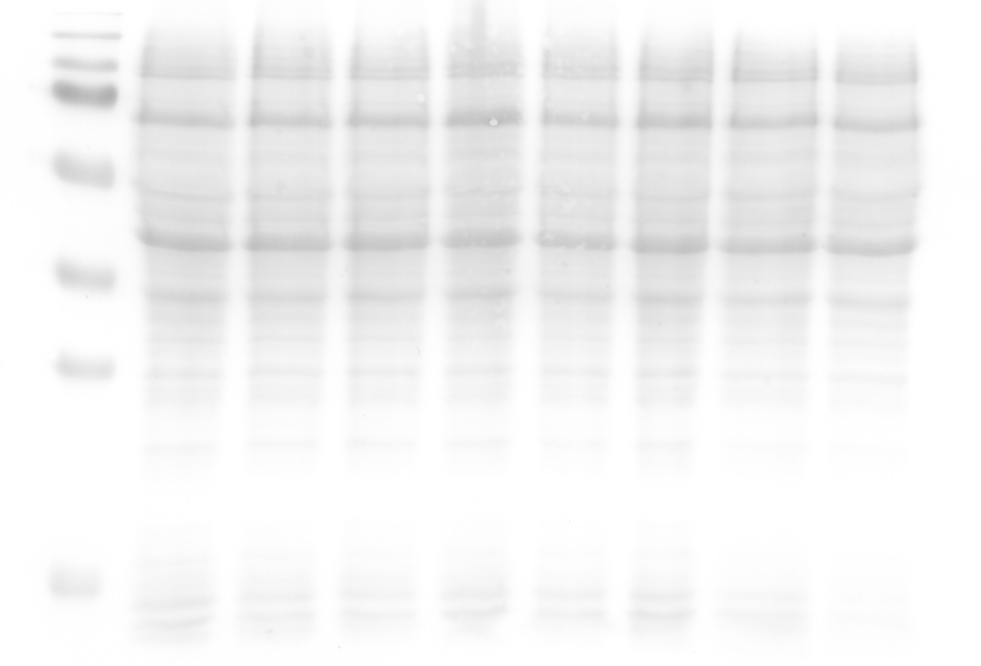

Supplement: Source data 1. [file elife-73944-data1.zip › Western_files/FigureS4_1D/dt37_rep1_ponceau.tif]

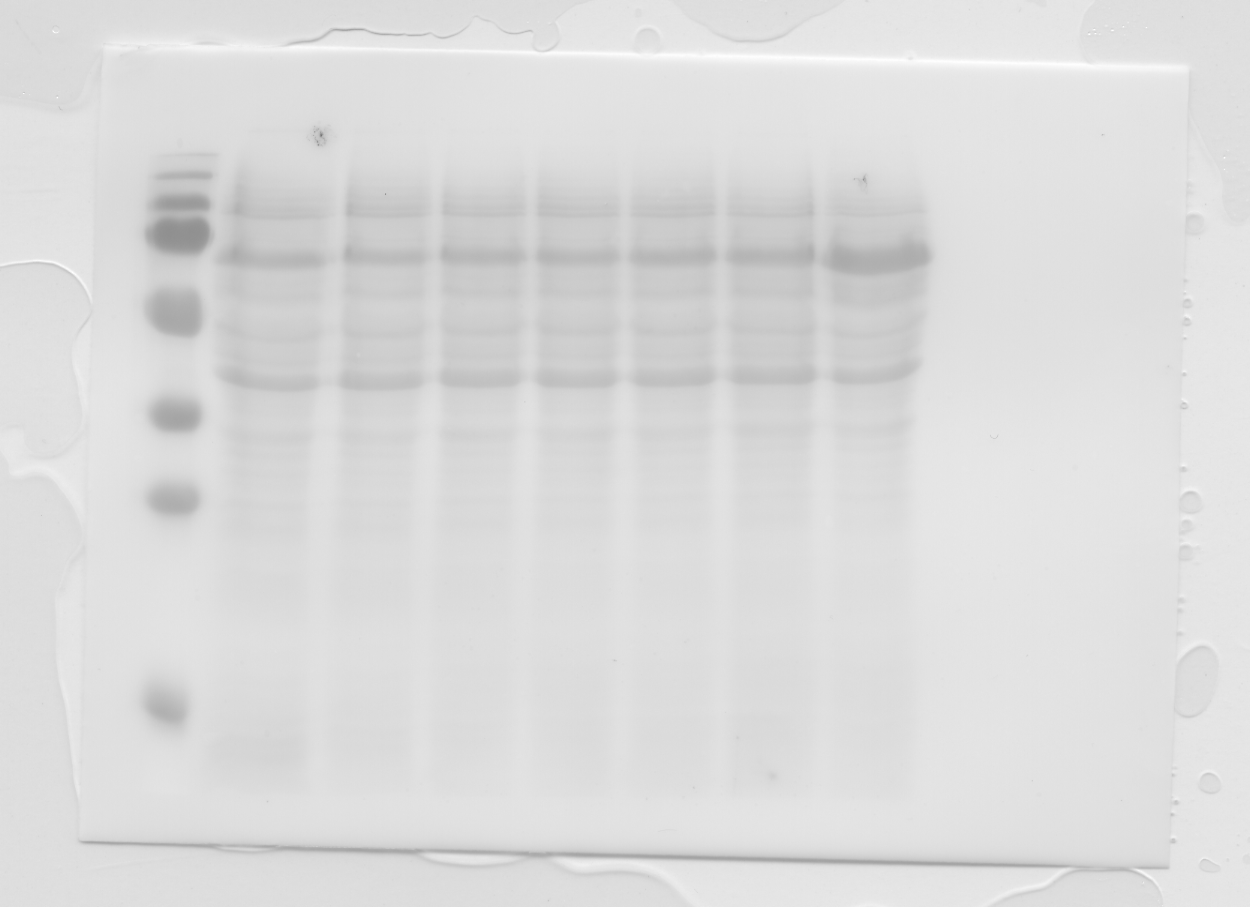

Supplement: Source data 1. [file elife-73944-data1.zip › Western_files/FigureS4_1D/wt_rep2_ponceau.tiff]

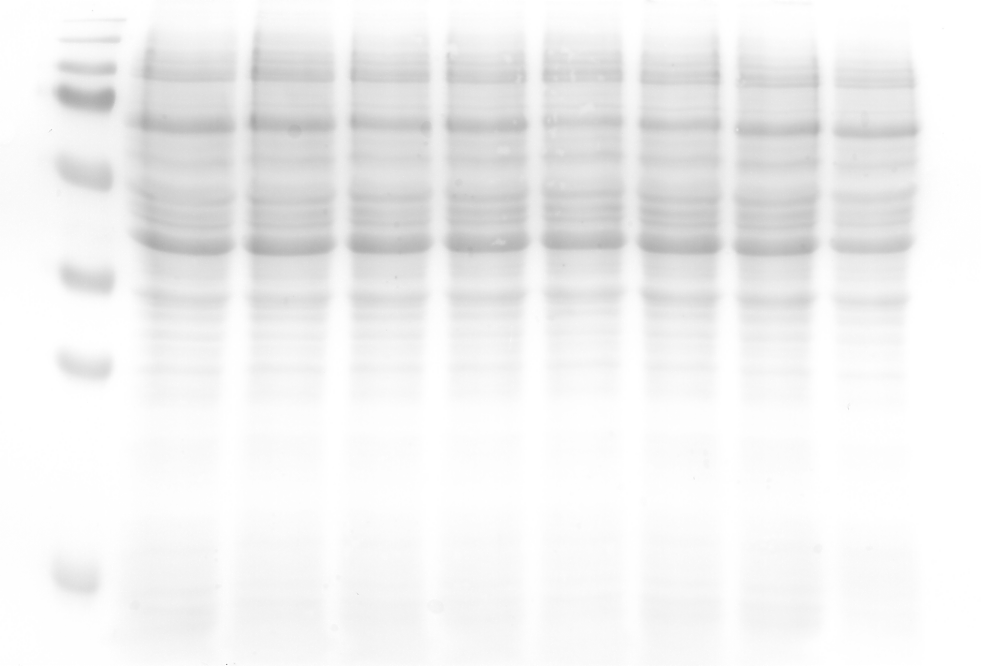

Supplement: Source data 1. [file elife-73944-data1.zip › Western_files/FigureS4_1D/dt37_rep3_ponceau.tif]

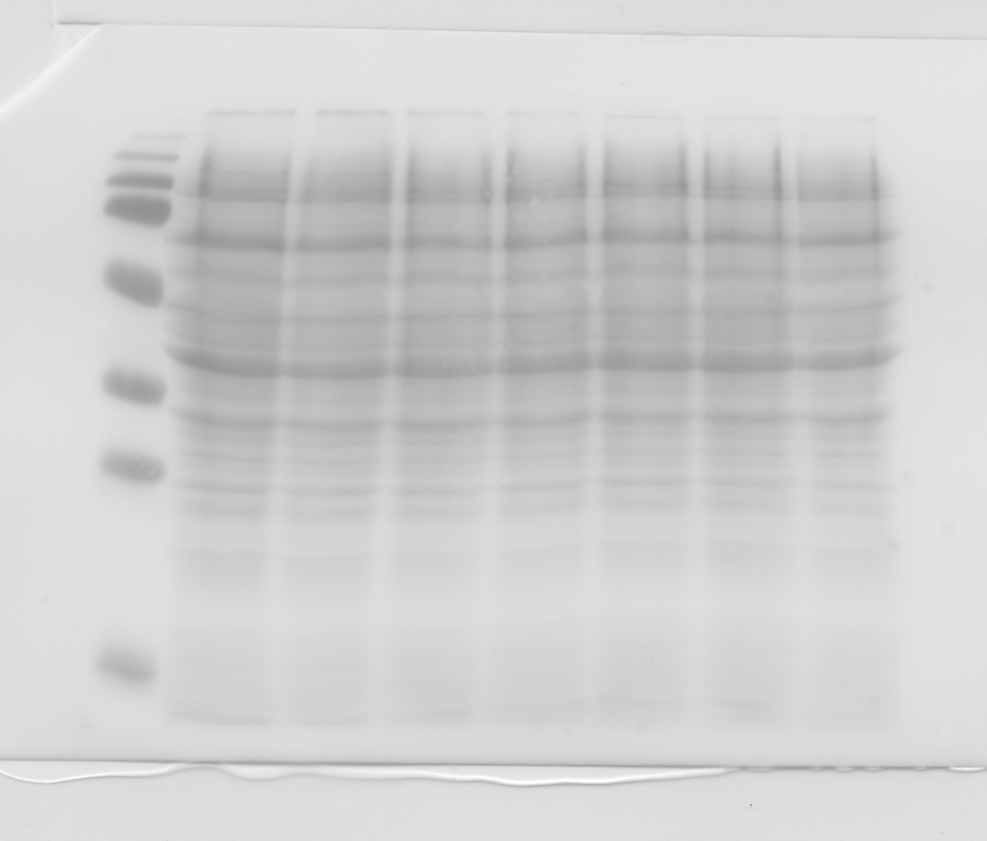

Supplement: Source data 1. [file elife-73944-data1.zip › Western_files/FigureS4_1D/wt_rep1_ponceau.tiff]

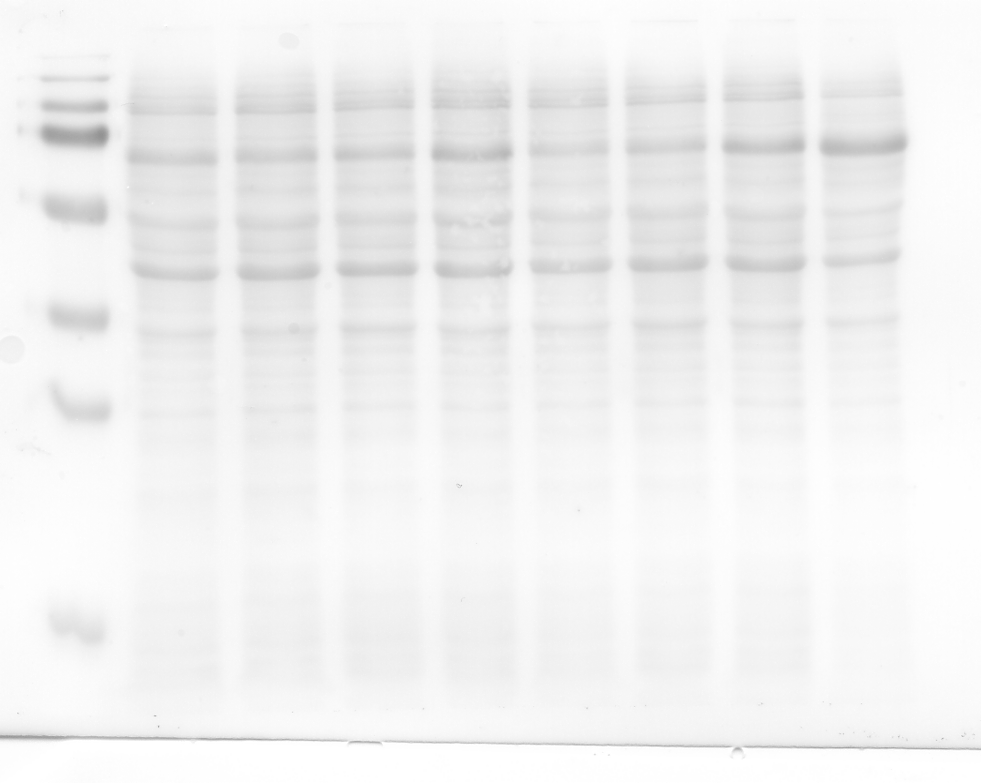

Supplement: Source data 1. [file elife-73944-data1.zip › Western_files/FigureS4_1D/dt37_ponceau_rep2.tif]

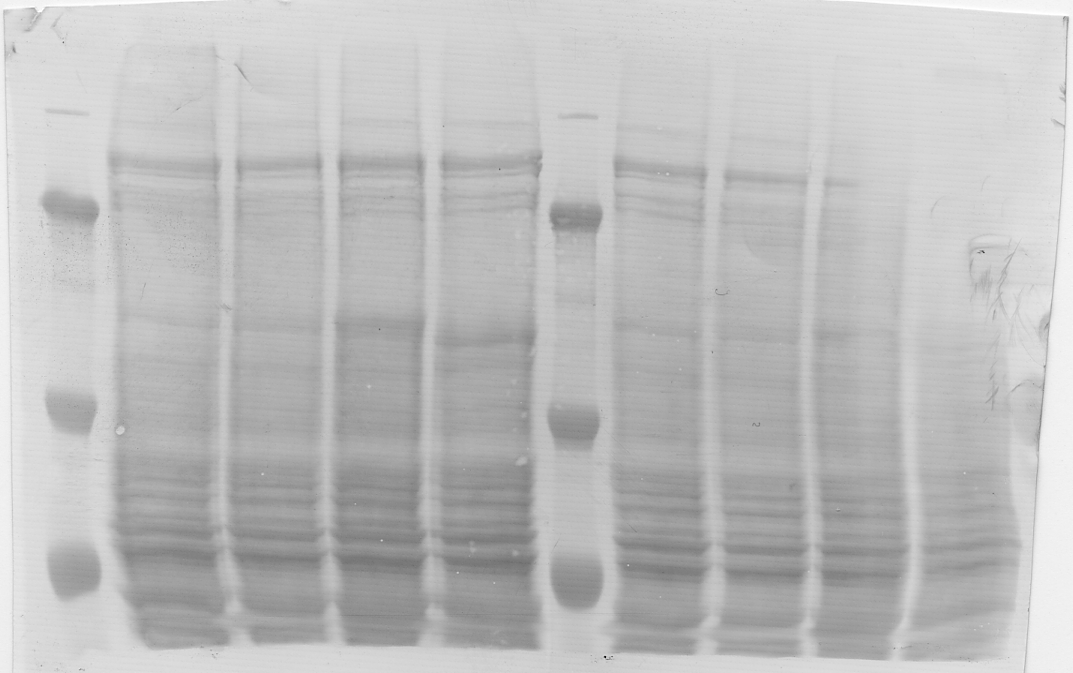

Supplement: Source data 1. [file elife-73944-data1.zip › Western_files/FigureS6_1B/rep3_PCNT_coomassie.tif]

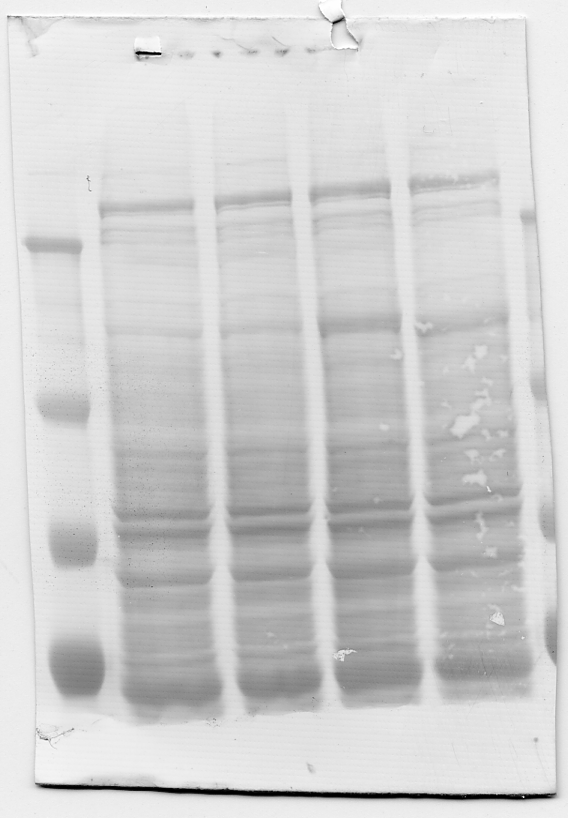

Supplement: Source data 1. [file elife-73944-data1.zip › Western_files/FigureS6_1B/rep3_CEP192_coomassie.tif]

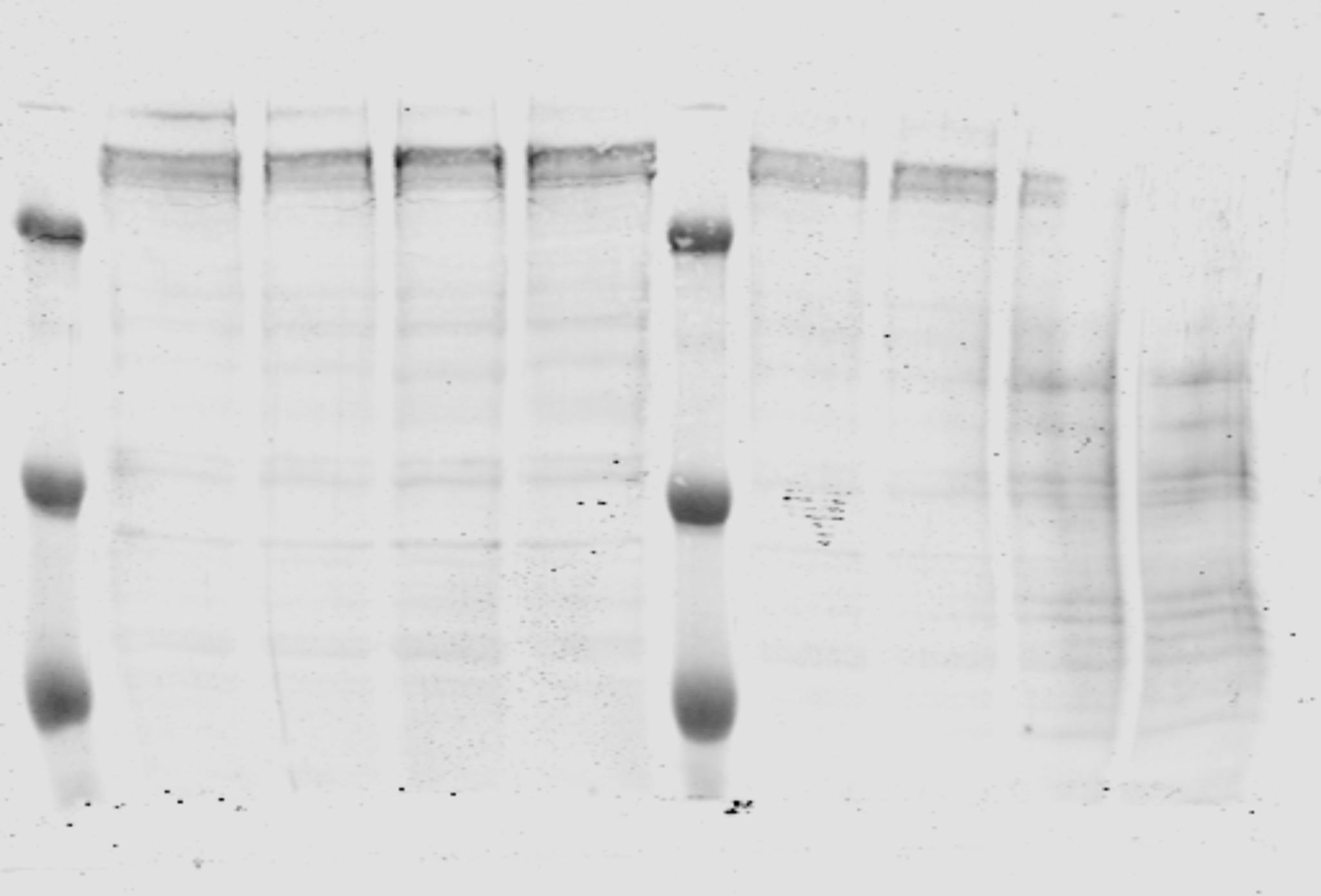

Supplement: Source data 1. [file elife-73944-data1.zip › Western_files/FigureS6_1B/rep3_PCNT.tif]

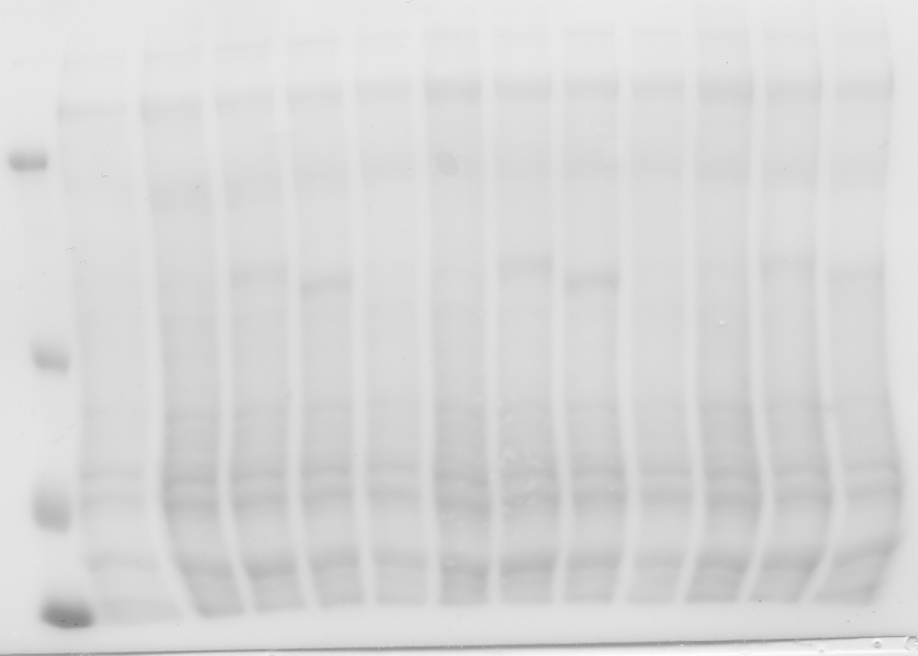

Supplement: Source data 1. [file elife-73944-data1.zip › Western_files/FigureS6_1B/rep1_rep2_PCNT_ponceau.tif]

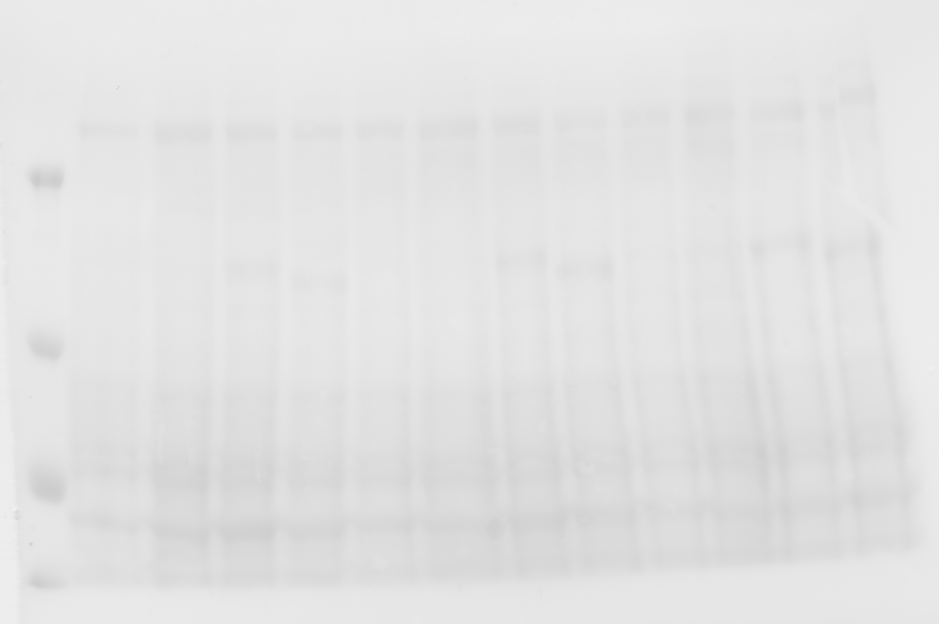

Supplement: Source data 1. [file elife-73944-data1.zip › Western_files/FigureS6_1B/rep1_rep2_CEP215_ponceau.tif]

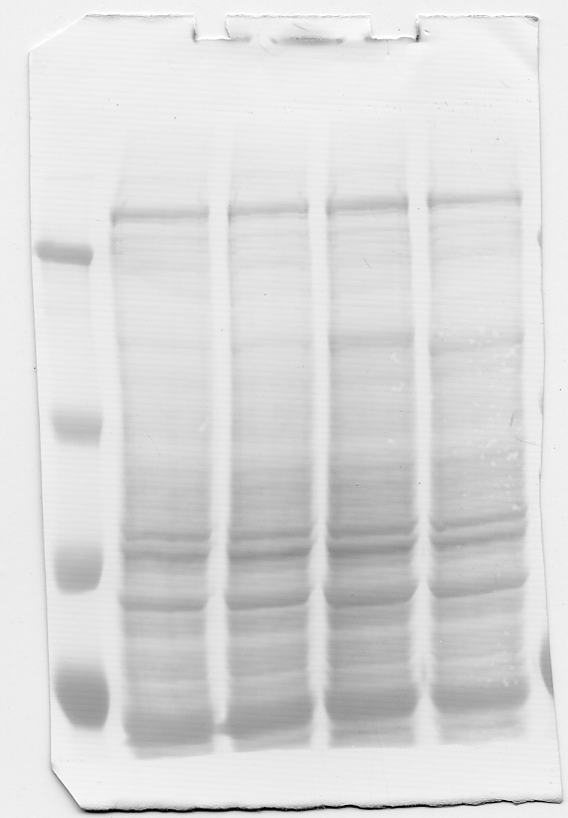

Supplement: Source data 1. [file elife-73944-data1.zip › Western_files/FigureS6_1B/rep3_CEP215_coomassie.tif]

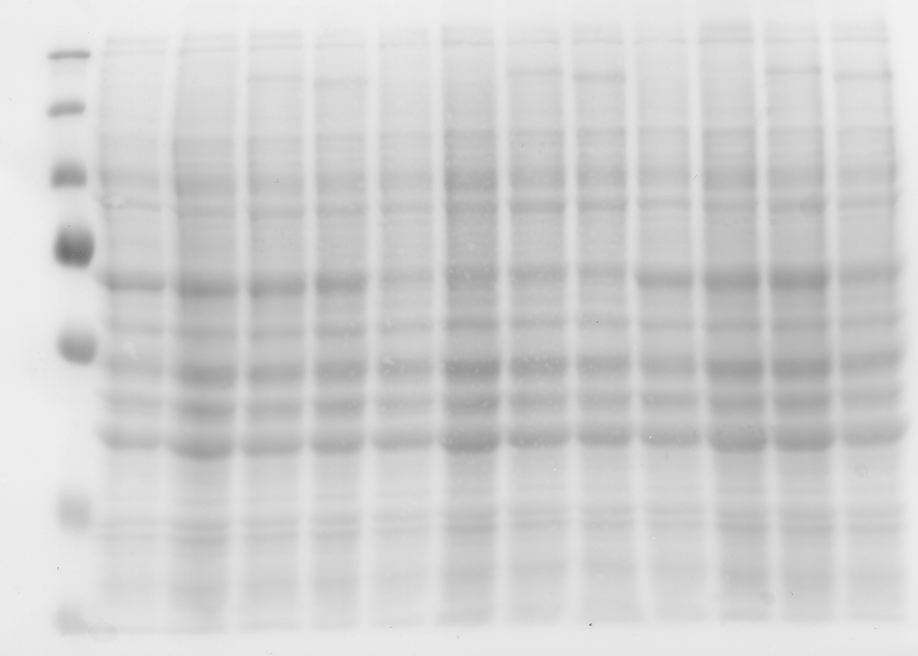

Supplement: Source data 1. [file elife-73944-data1.zip › Western_files/FigureS6_1B/FLAG_TRIM37_ponceau.tif]

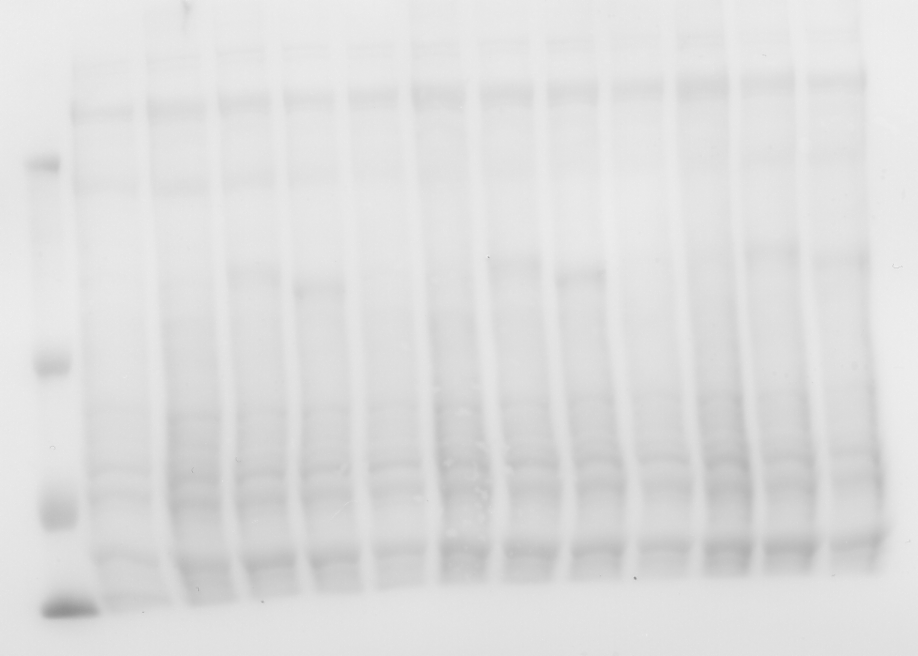

Supplement: Source data 1. [file elife-73944-data1.zip › Western_files/FigureS6_1B/rep1_rep2_CEP192_ponceau.tif]

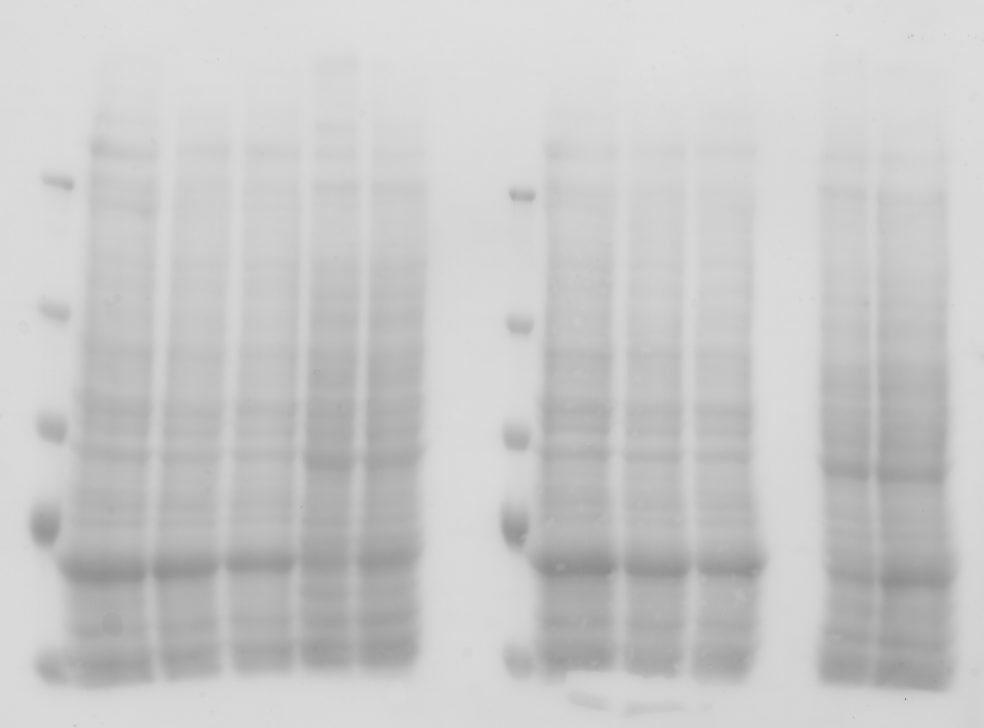

Supplement: Source data 1. [file elife-73944-data1.zip › Western_files/FigureS3_1C/ponceau.tiff]

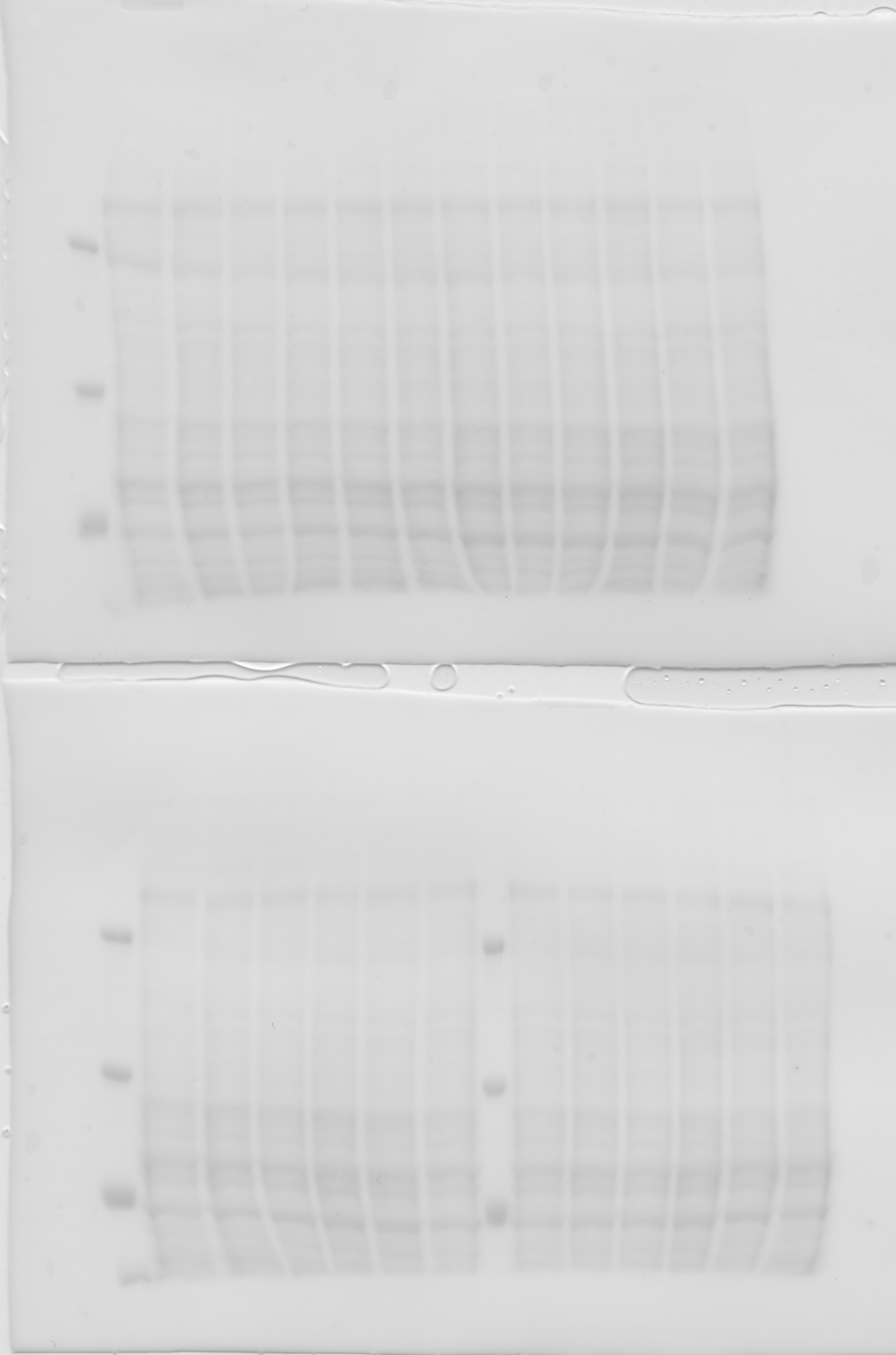

Supplement: Source data 1. [file elife-73944-data1.zip › Western_files/Figure6B/rep3_ponceau_top.tiff]

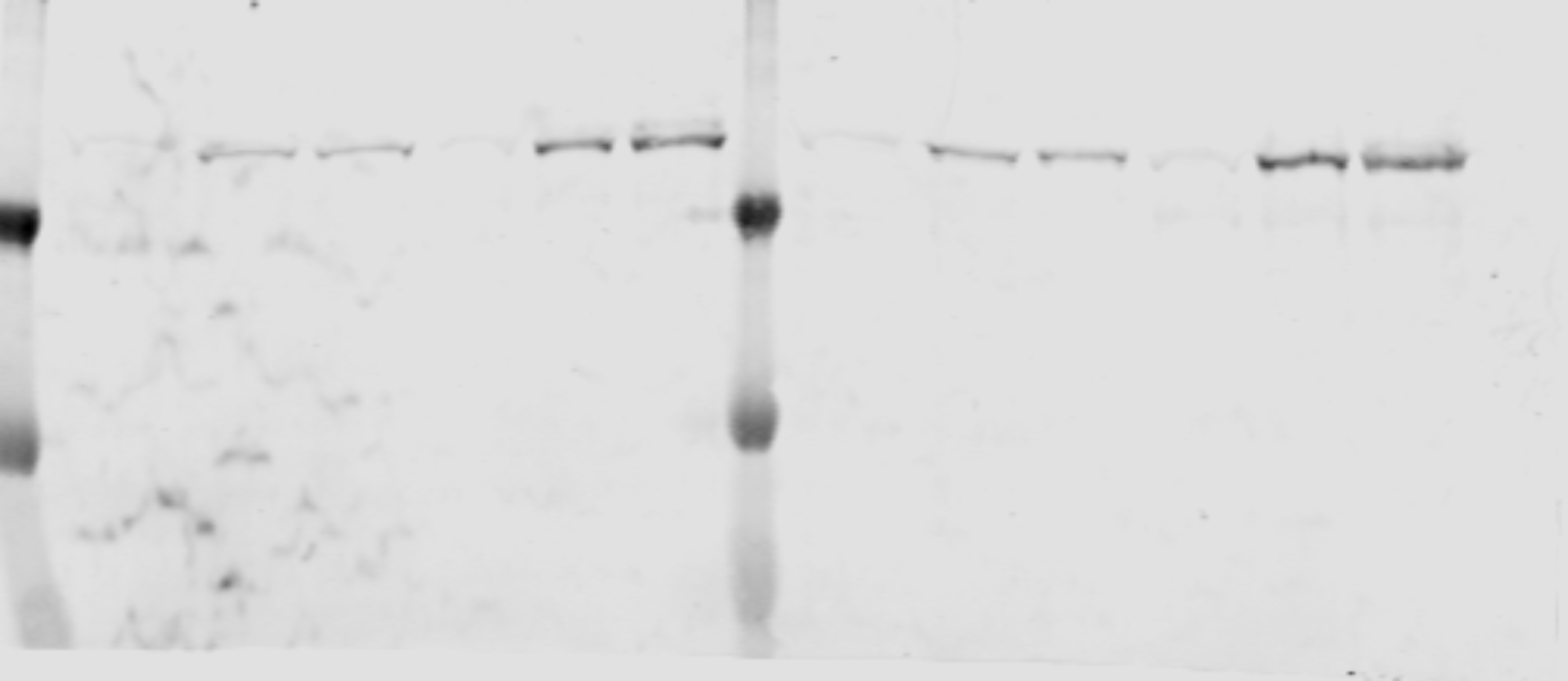

Supplement: Source data 1. [file elife-73944-data1.zip › Western_files/Figure6B/rep1_TRIM37.tif]

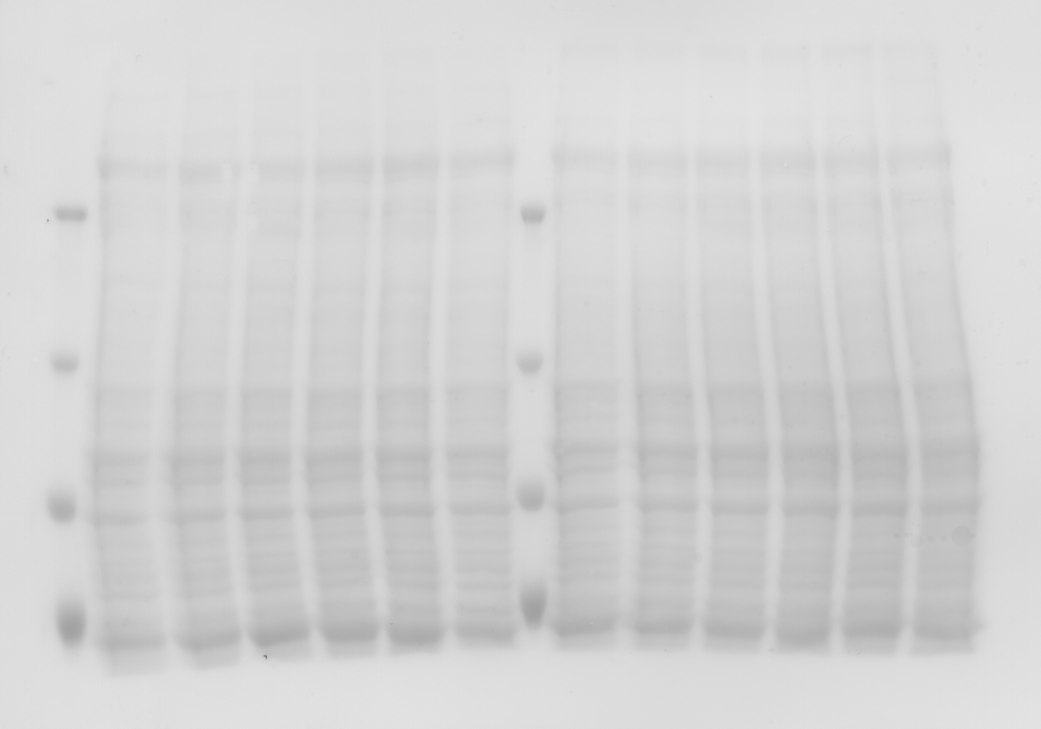

Supplement: Source data 1. [file elife-73944-data1.zip › Western_files/Figure6B/rep1_ponceau.tiff]

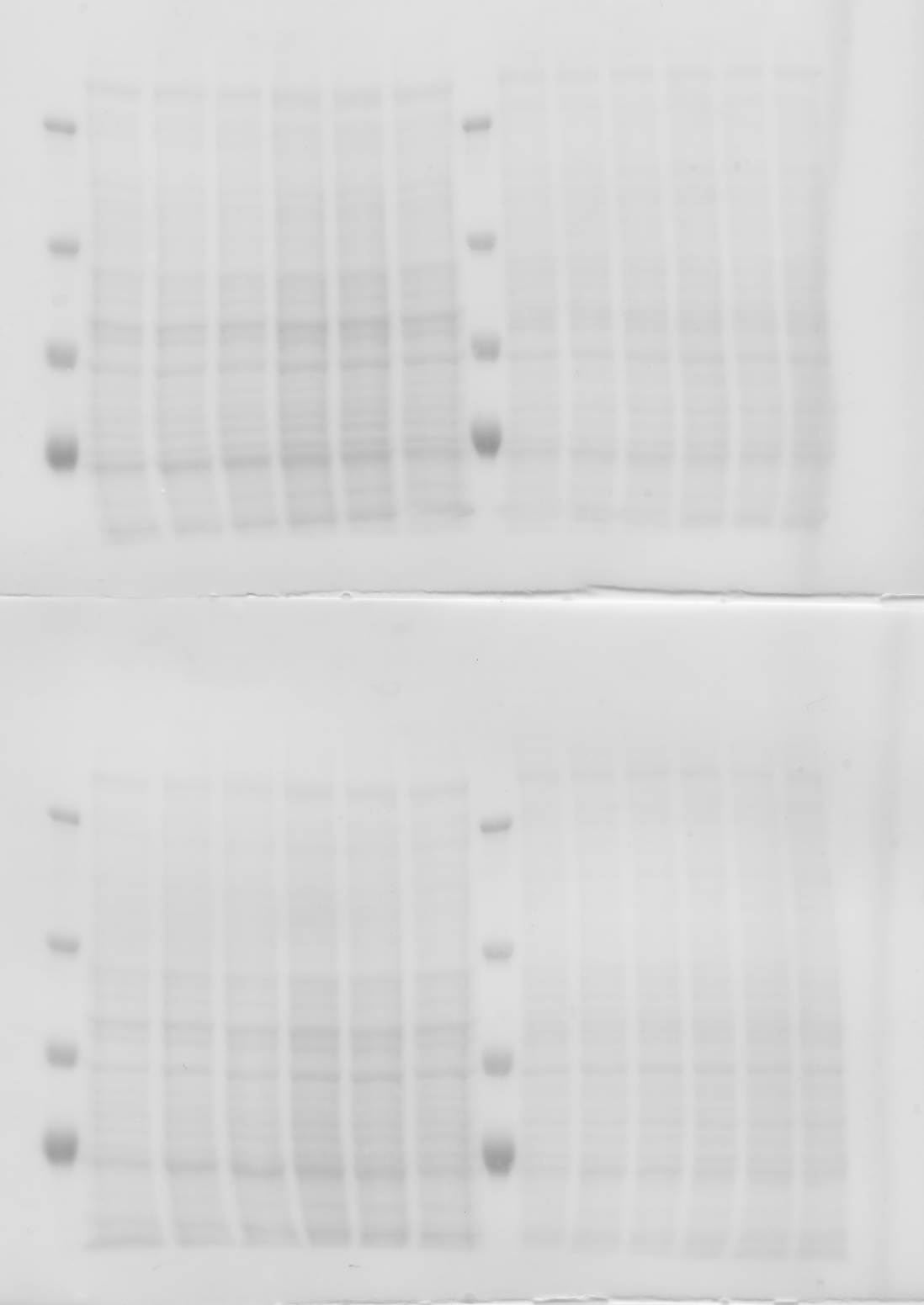

Supplement: Source data 1. [file elife-73944-data1.zip › Western_files/Figure6B/rep2_ponceau_top.tiff]

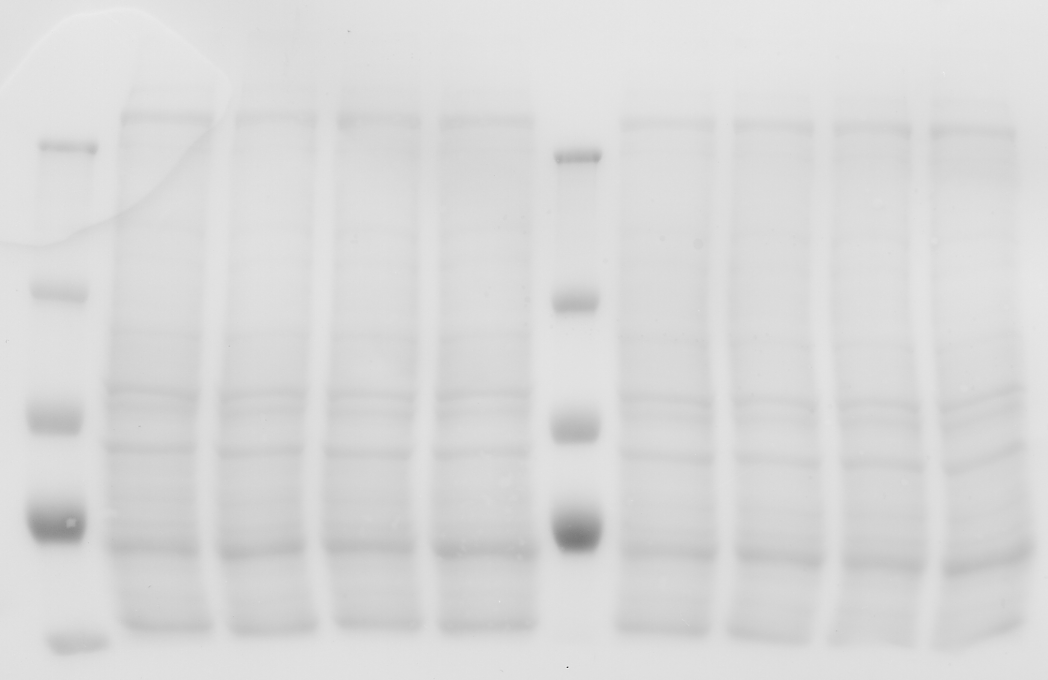

Supplement: Source data 1. [file elife-73944-data1.zip › Western_files/FigureS4_3C/rep3_ponceau_left.tif]

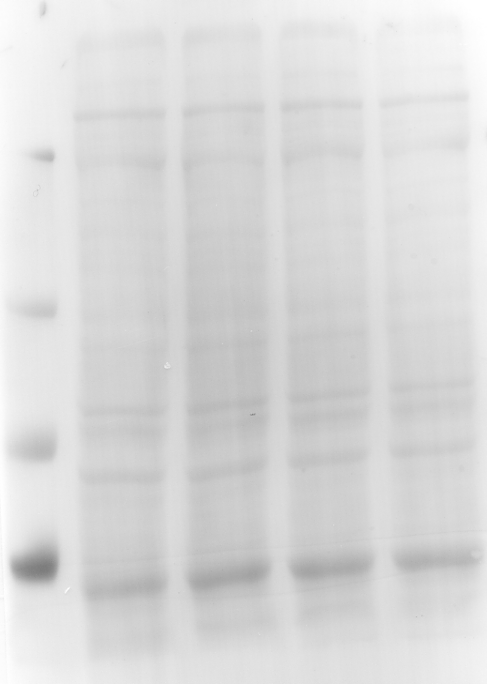

Supplement: Source data 1. [file elife-73944-data1.zip › Western_files/FigureS4_3C/rep2_ponceau.tif]

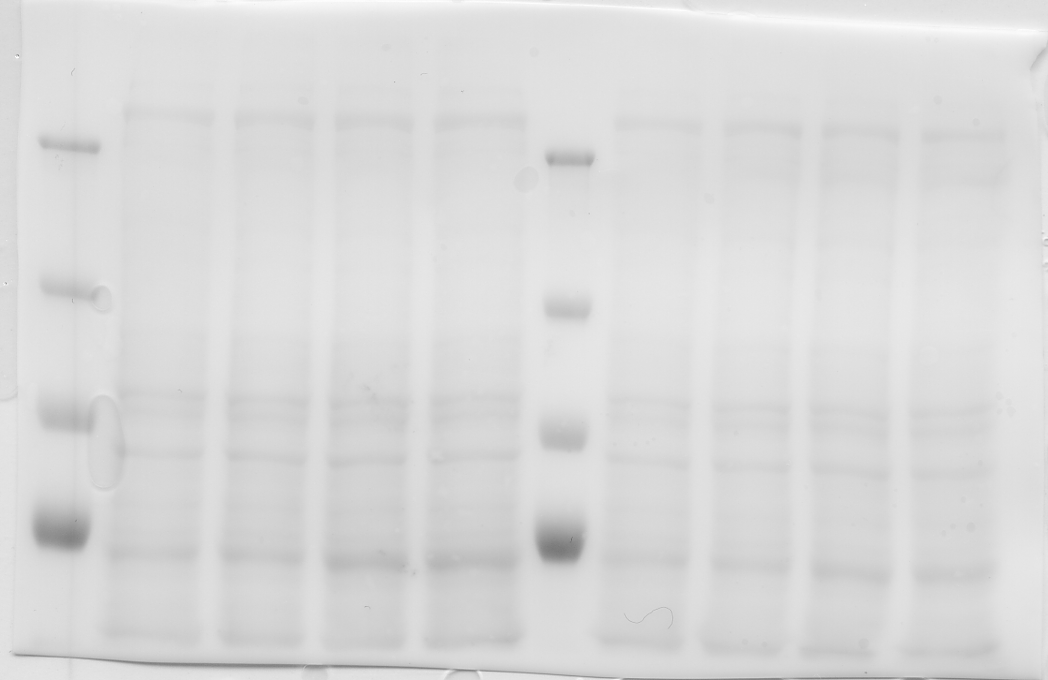

Supplement: Source data 1. [file elife-73944-data1.zip › Western_files/FigureS4_3C/rep1_ponceau_left.tif]

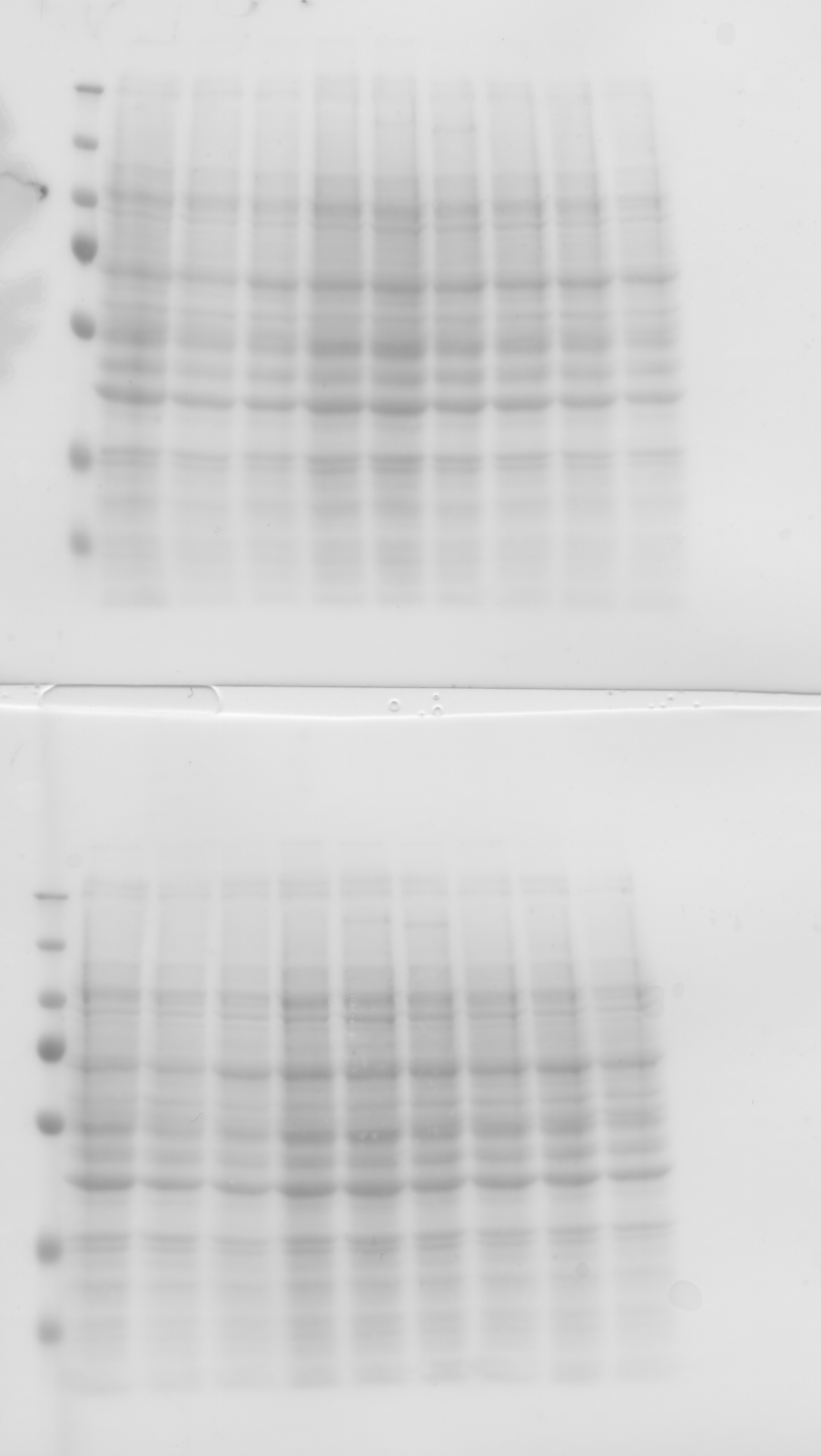

Supplement: Source data 1. [file elife-73944-data1.zip › Western_files/FigureS4_1A/ponceau_top.tiff]

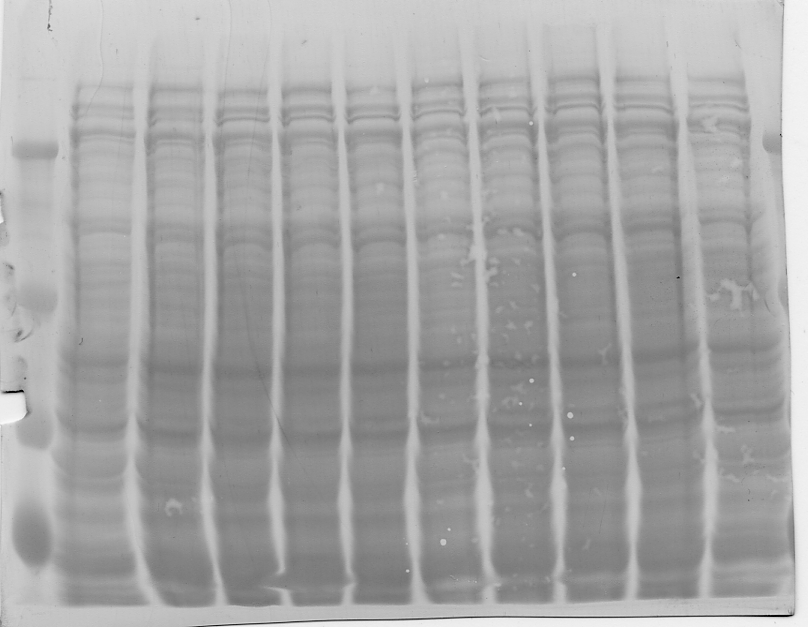

Supplement: Source data 1. [file elife-73944-data1.zip › Western_files/Figure7F/coomassie.tif]

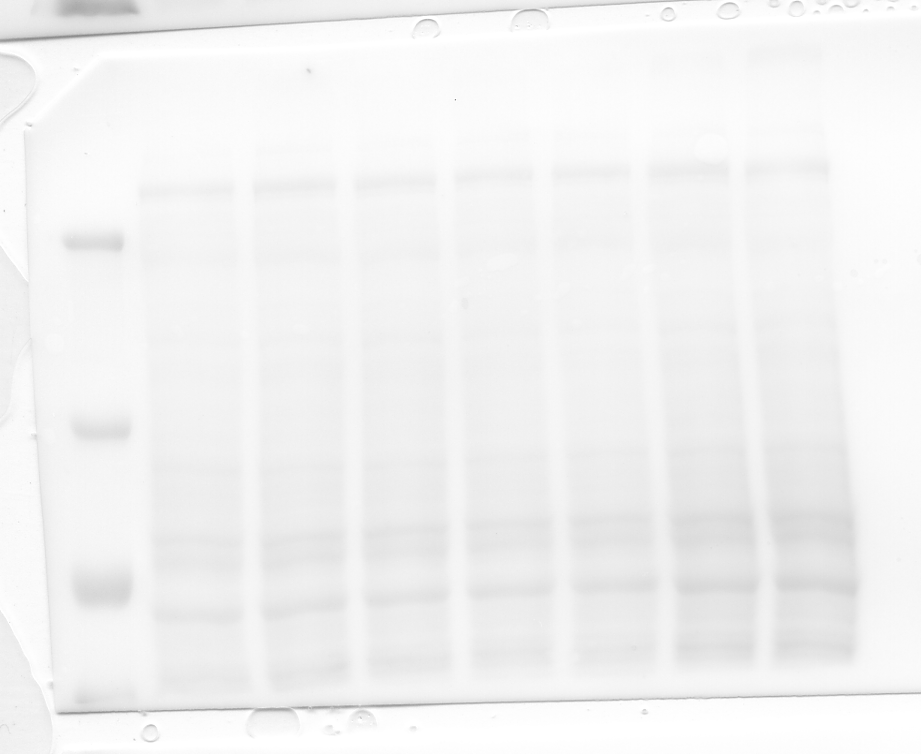

Supplement: Source data 1. [file elife-73944-data1.zip › Western_files/FigureS4_2A/rep1_ponceau.tif]

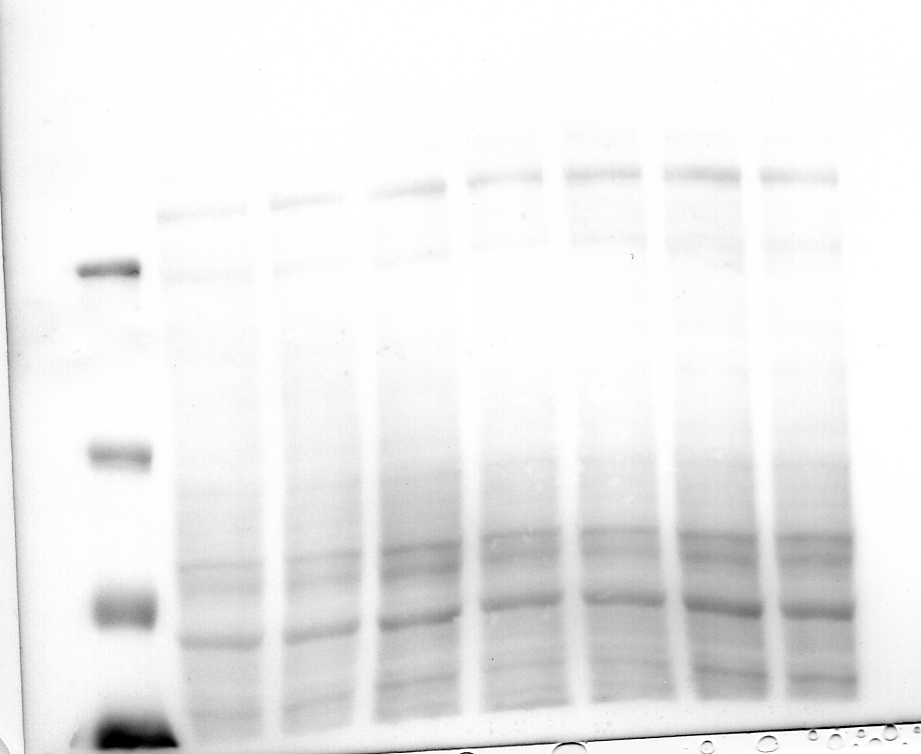

Supplement: Source data 1. [file elife-73944-data1.zip › Western_files/FigureS4_2A/rep2_ponceau.tif]

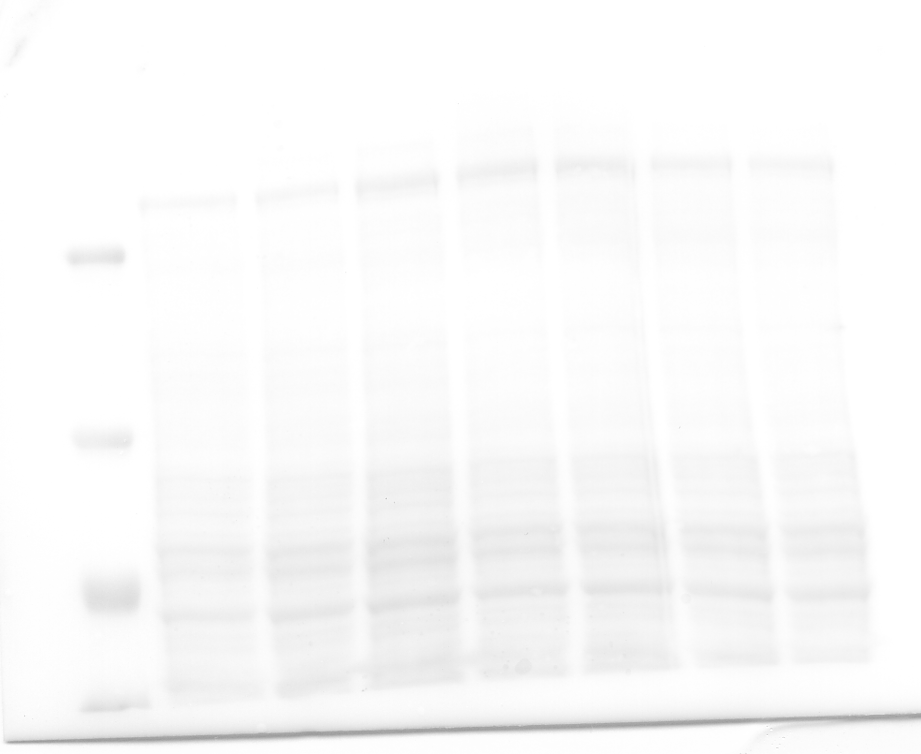

Supplement: Source data 1. [file elife-73944-data1.zip › Western_files/FigureS4_2A/rep3_ponceau.tif]

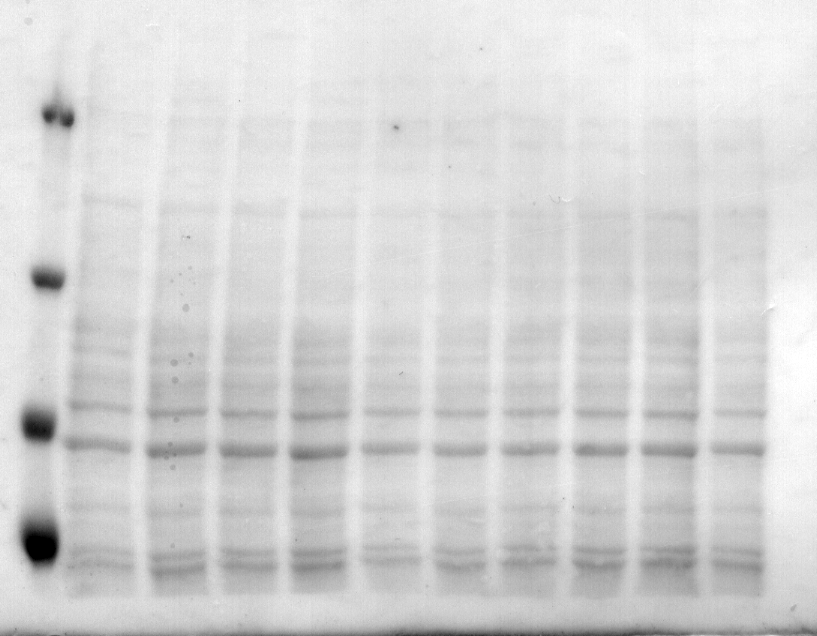

Supplement: Source data 1. [file elife-73944-data1.zip › Western_files/Figure7G/ponceau.tiff]

1B

p53

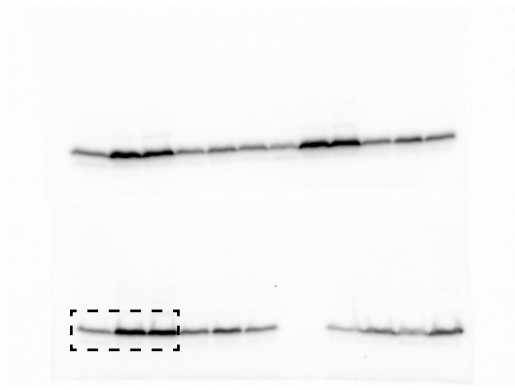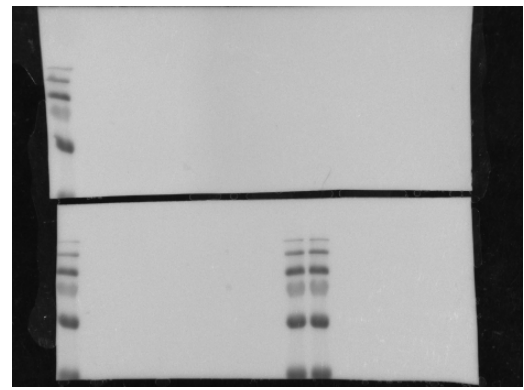

p21

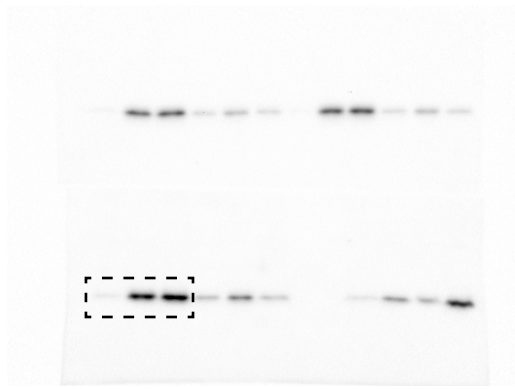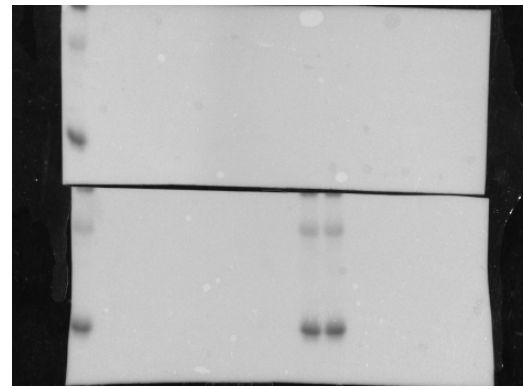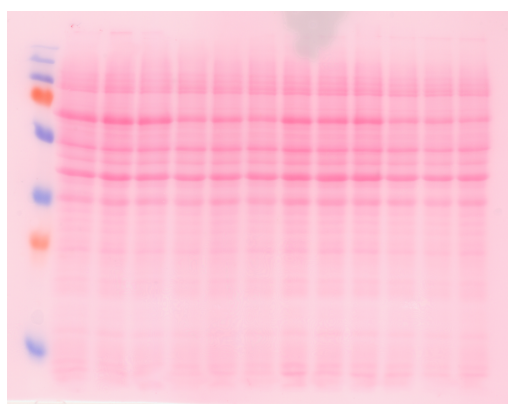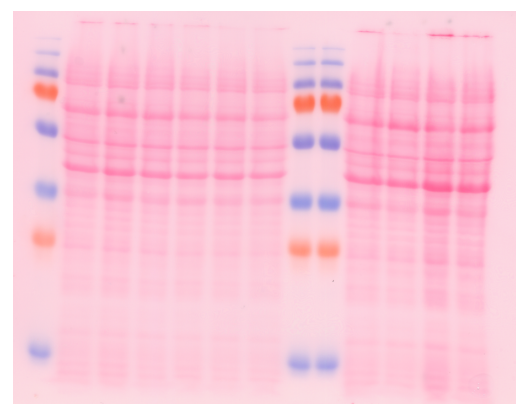

Figure1 Western blots

\*\*\*samples imaged with those for Figure 3

Supplement: Source data 2. — (.zip file). [file elife-73944-data2.zip › TkachJM_source_Western/Figure1_western.pdf]

7B

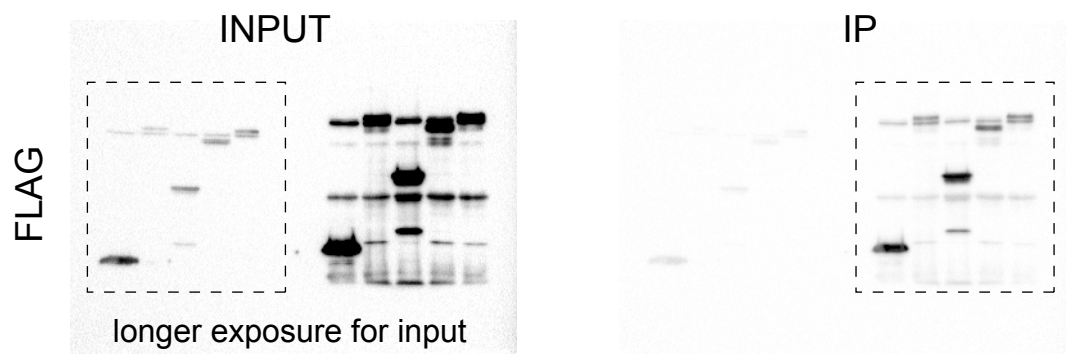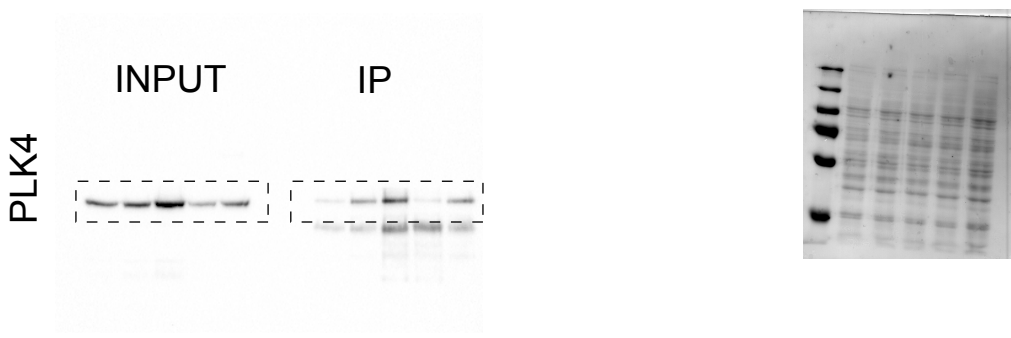

7D

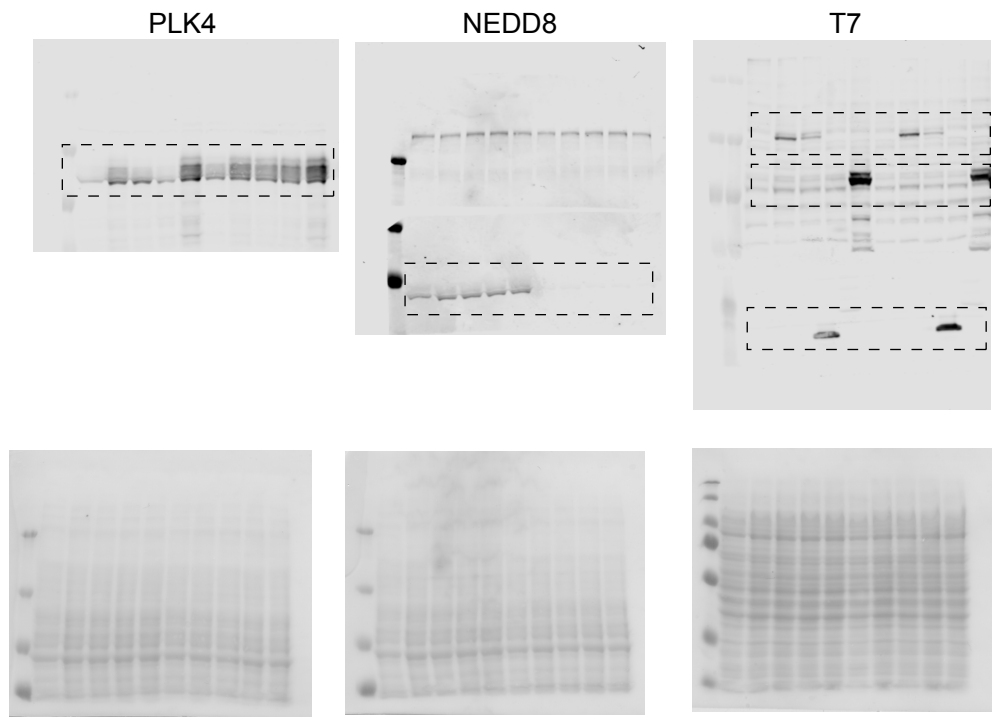

Figure 7 Western blots

7E

PLK4

HA-Ub

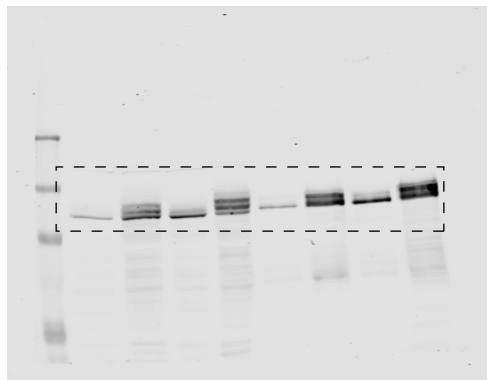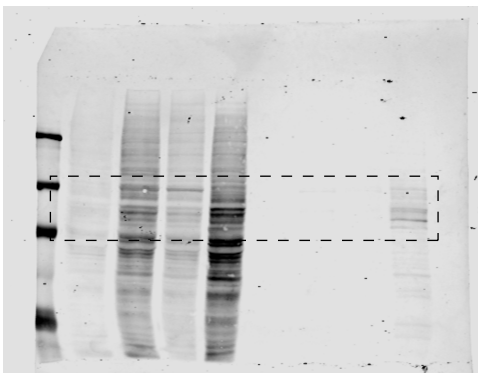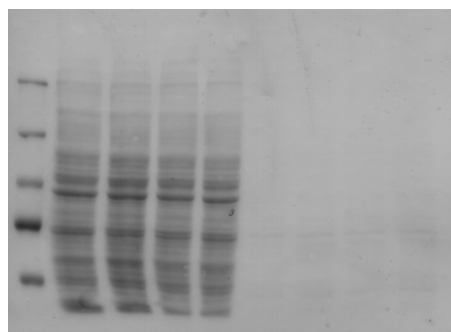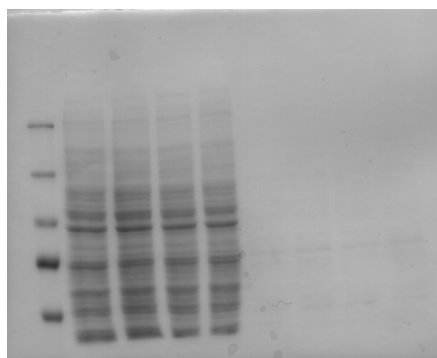

7F

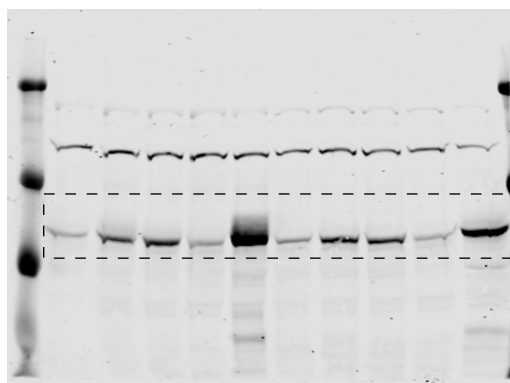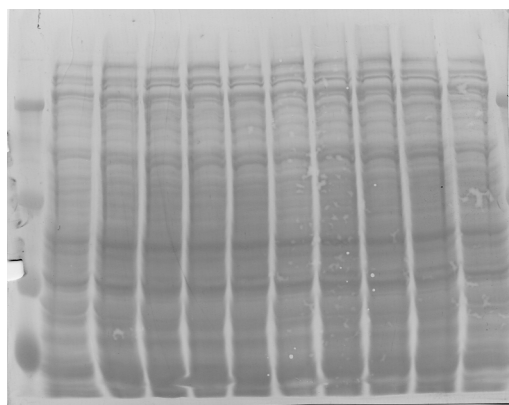

Figure 7 Western blots

7G

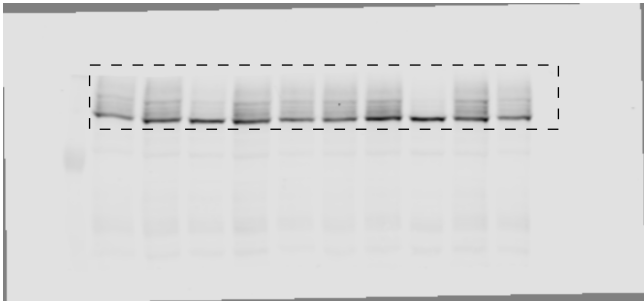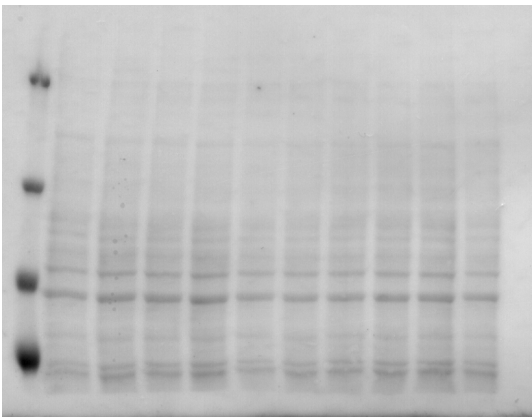

Figure 7 Western blots

Supplement: Source data 2. — (.zip file). [file elife-73944-data2.zip › TkachJM_source_Western/Figure7_western.pdf]

1C

TRIM37

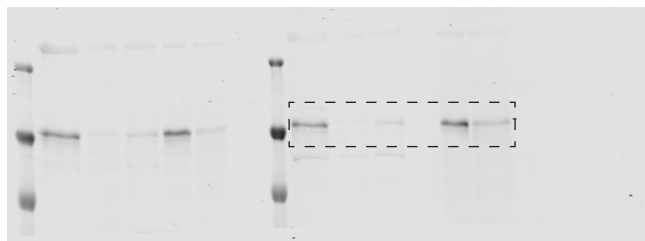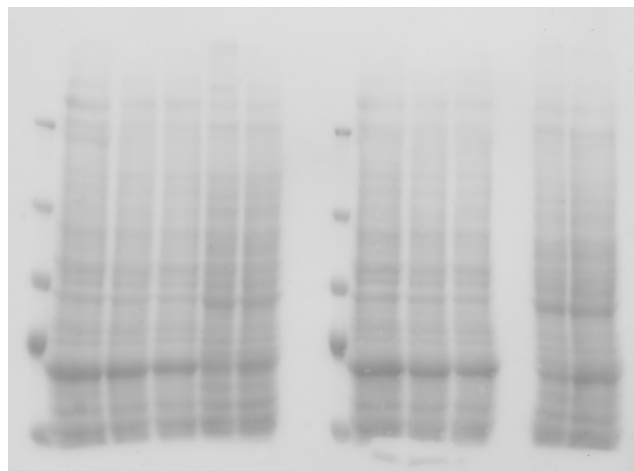

2A

FLAG

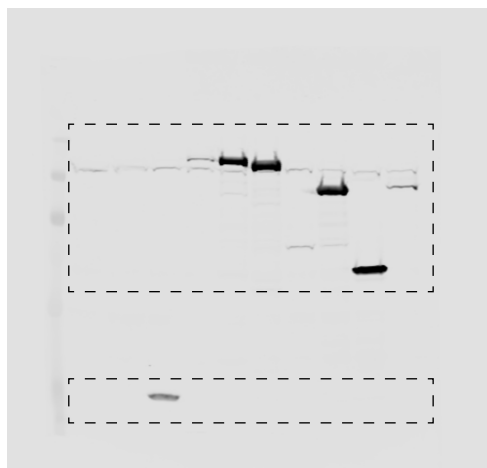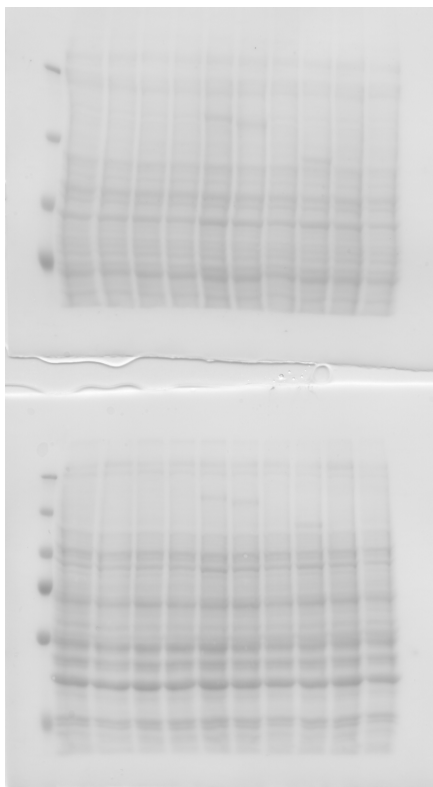

Figure 3 - figure supplement 1 and 2 Western blots

Supplement: Source data 2. — (.zip file). [file elife-73944-data2.zip › TkachJM_source_Western/Figure3_supp_western.pdf]

3B

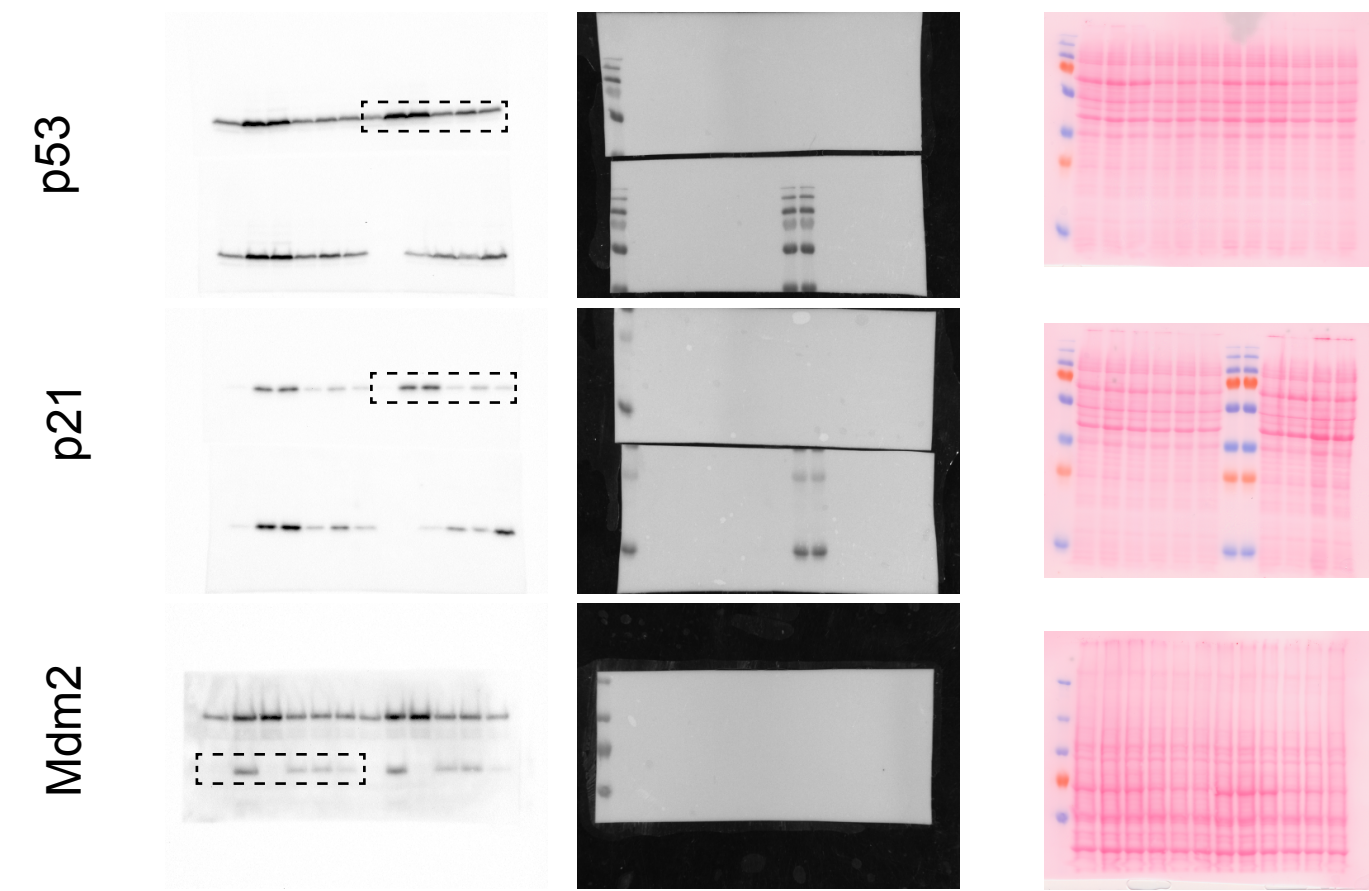

Figure 3 Western blots

\*\*\*samples imaged with those from Figure 1

Supplement: Source data 2. — (.zip file). [file elife-73944-data2.zip › TkachJM_source_Western/Figure3_western.pdf]

6B

CEP192

TRIM37

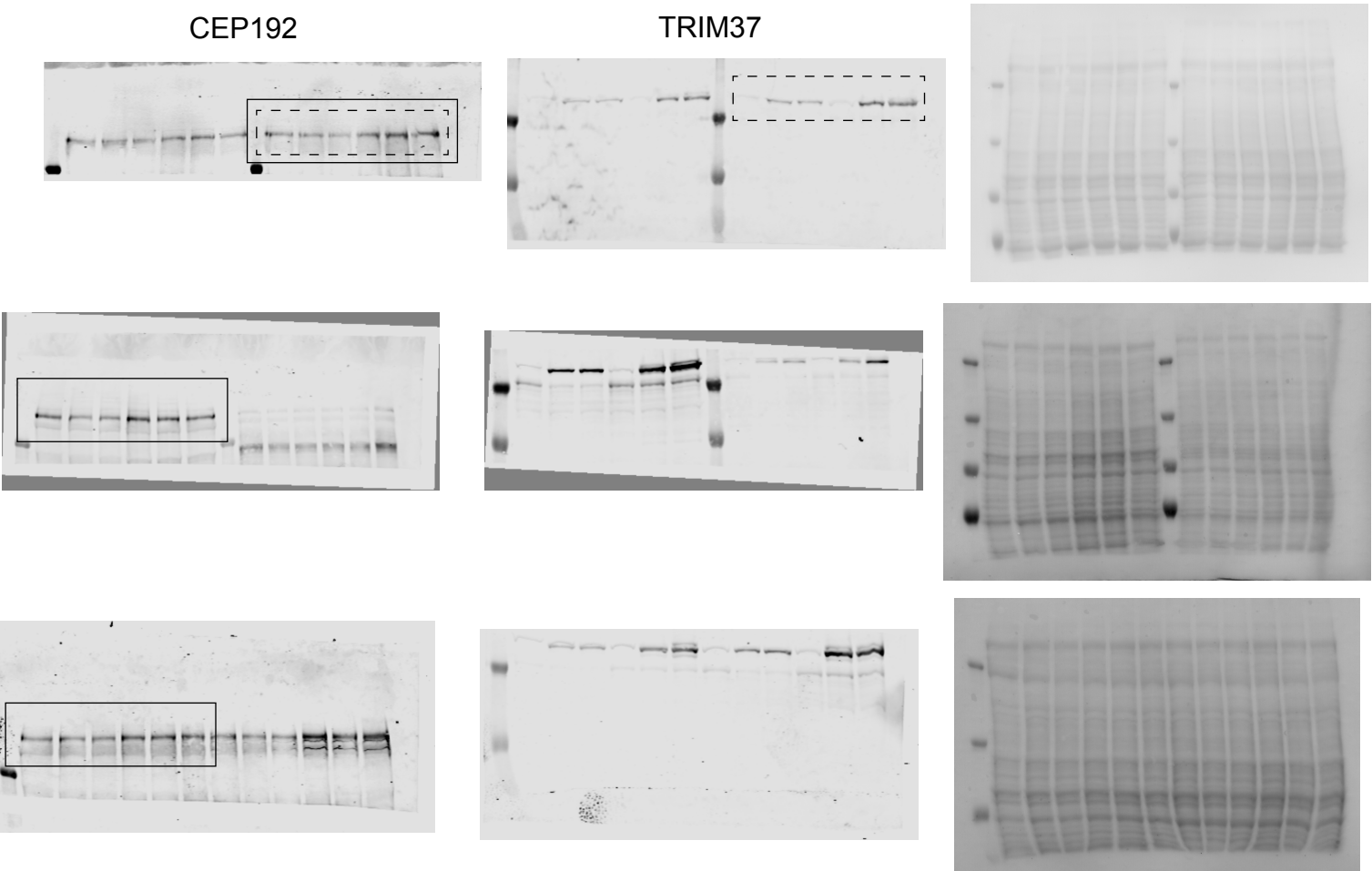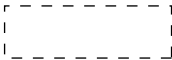

used for figure

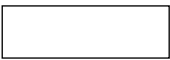

used for quantification

Figure 6 Western blots

Supplement: Source data 2. — (.zip file). [file elife-73944-data2.zip › TkachJM_source_Western/Figure6_Western.pdf]
