# Supplementary material for: Global cellular response to chemical perturbation of PLK4 activity and abnormal centrosome number
Source: eLife. 2022 Jun 27;11:e73944. doi: 10.7554/eLife.73944 (PMC9236612; doi:10.7554/eLife.73944)
Supplement: Source data 2. — (.zip file). [file elife-73944-data2.zip › TkachJM_source_Western/Figure4_supp_western.pdf]

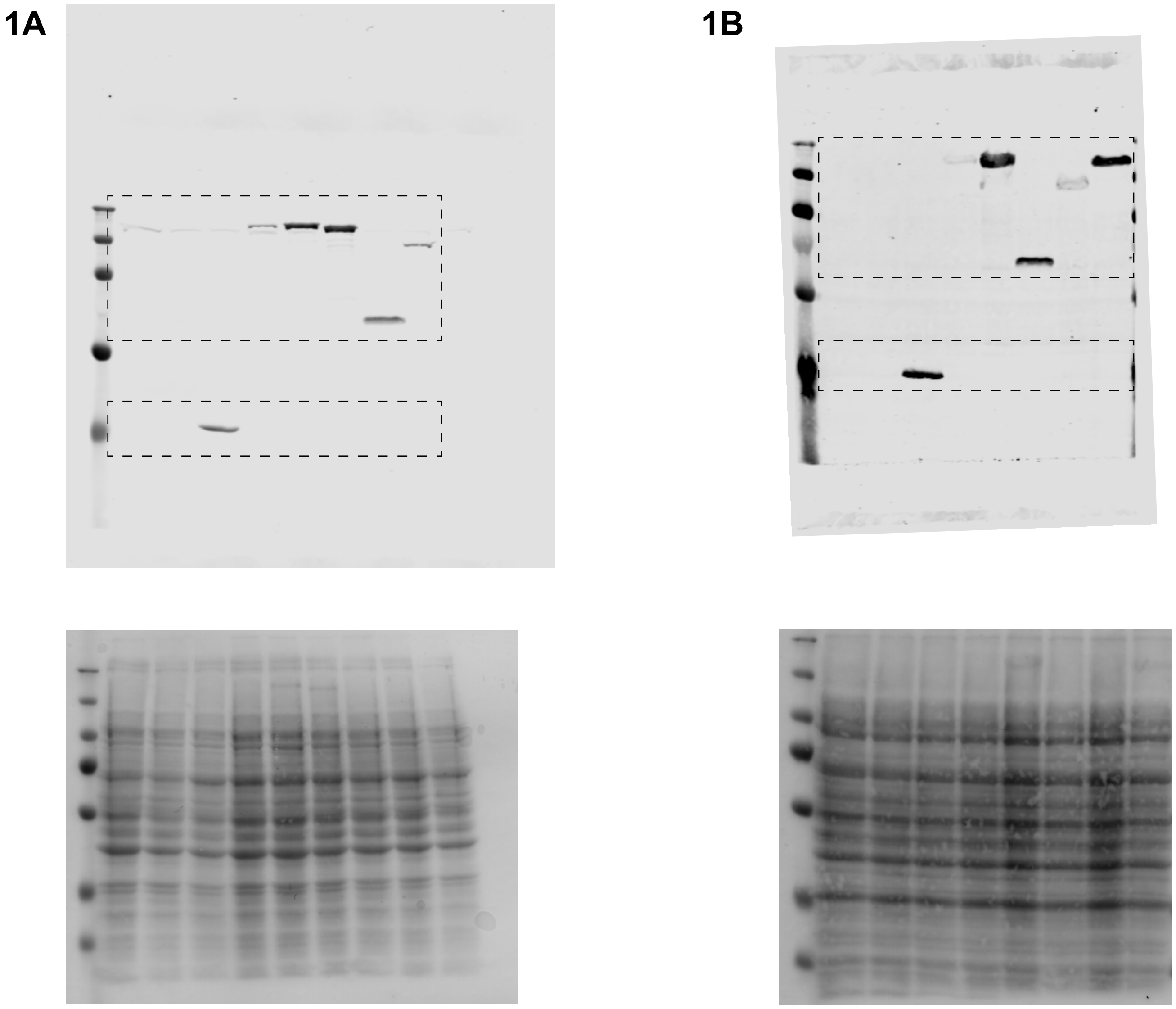

Figure 4 - figure supplement 1 Western blots

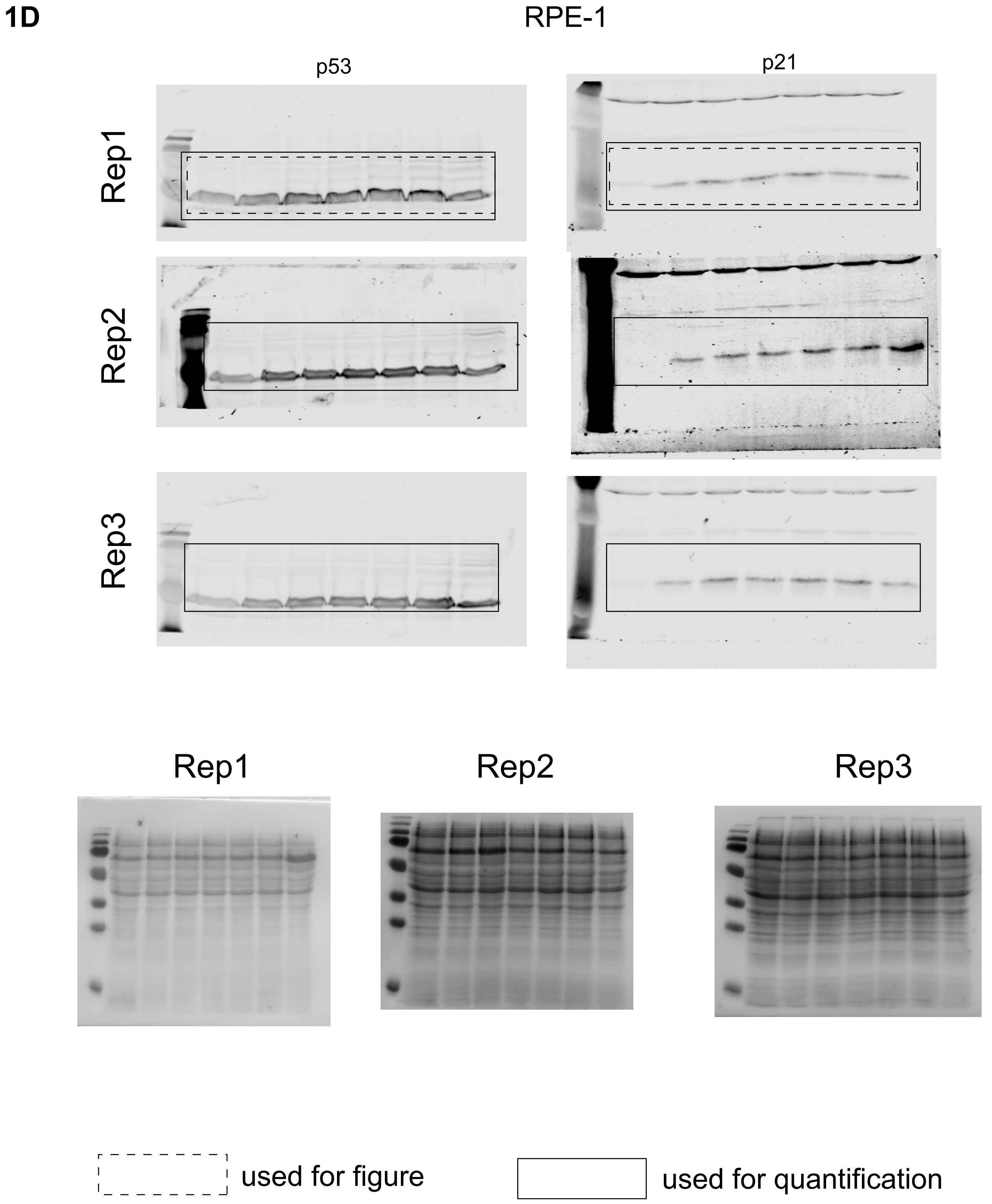

Figure 4 - figure supplement 1 Western blots

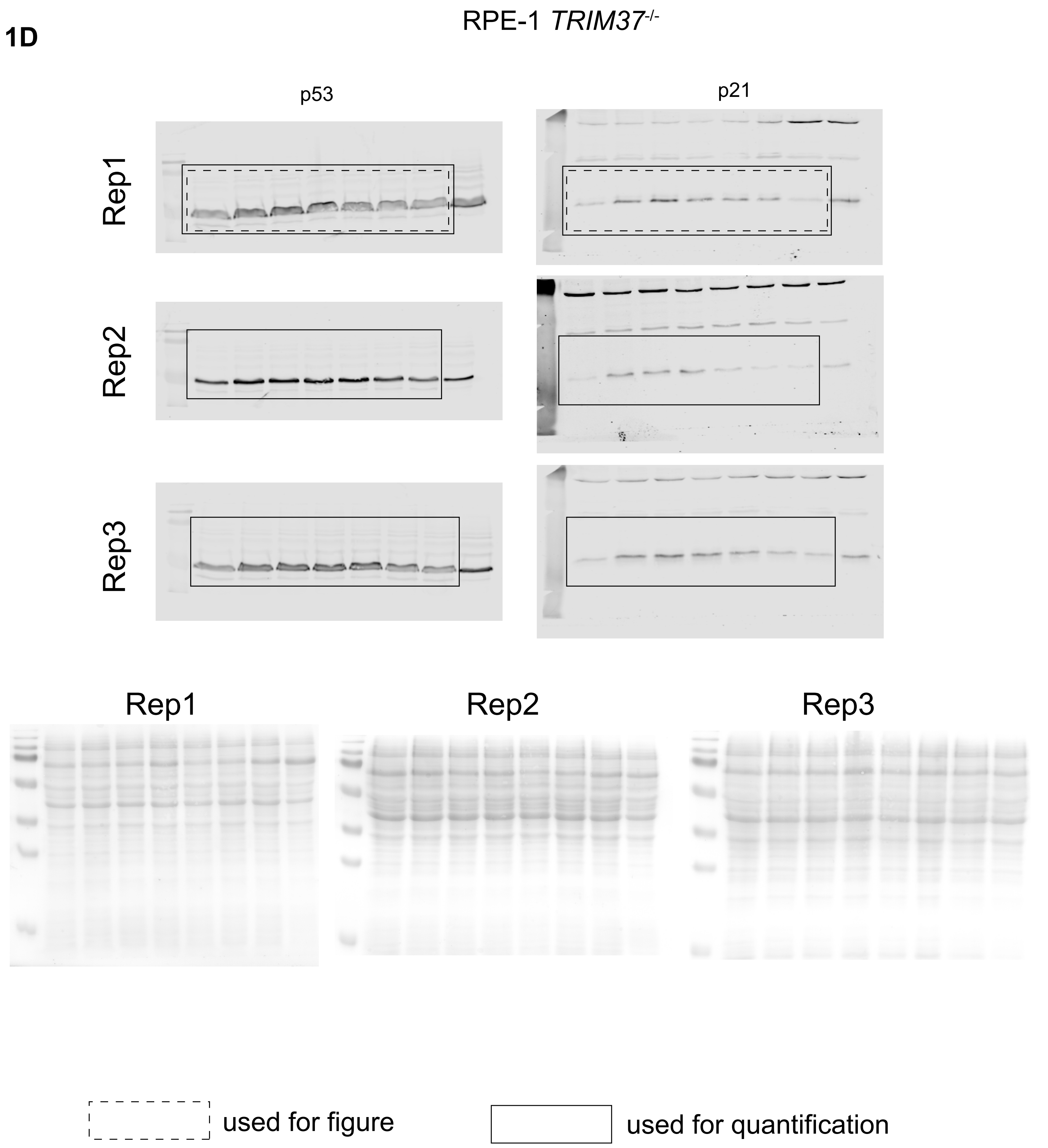

Figure 4 - figure supplement 1 Western blots

2A

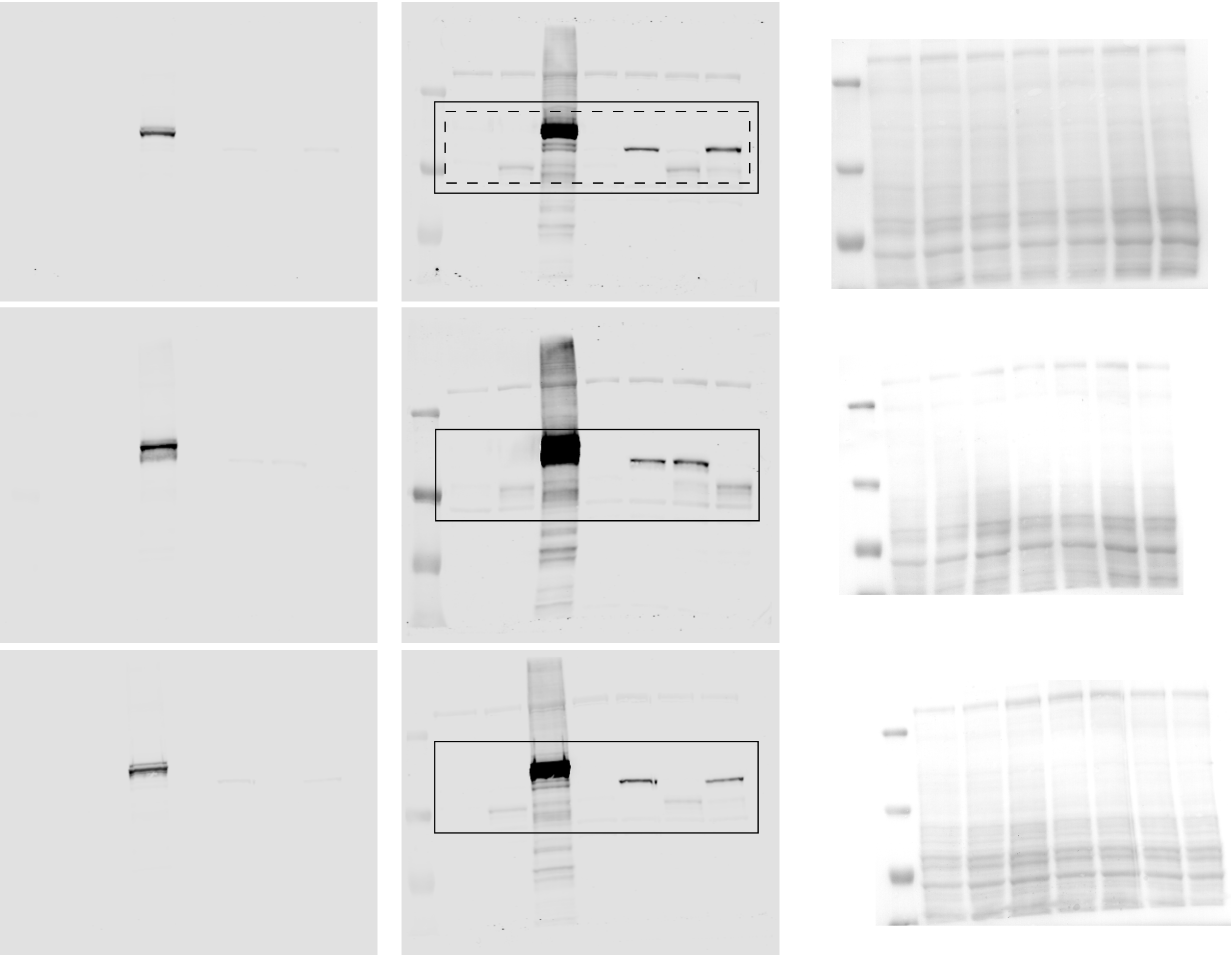

(over-saturated)

used for figure (dashed box) | used for quantification (solid box)

Figure 4 - figure supplement 2 Western blots

2D

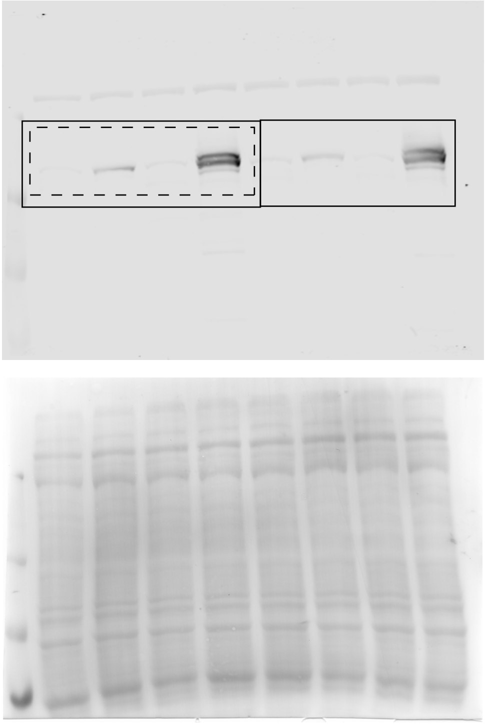

3A

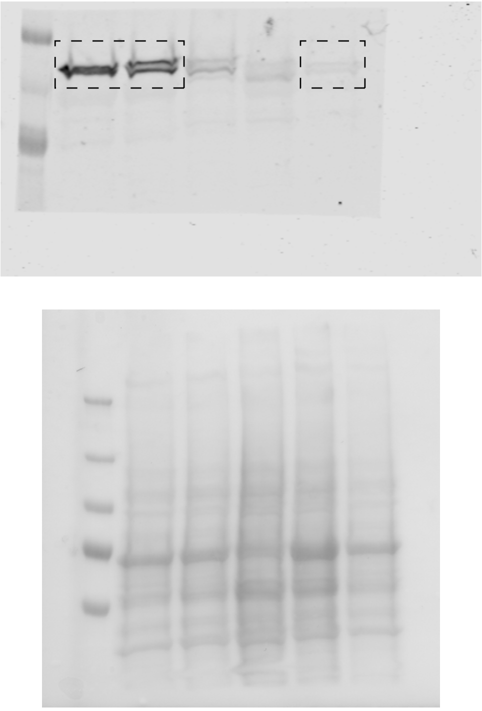

used for figure

used for quantification

Figure 4 - figure supplement 2 and 3 Western blots

3C

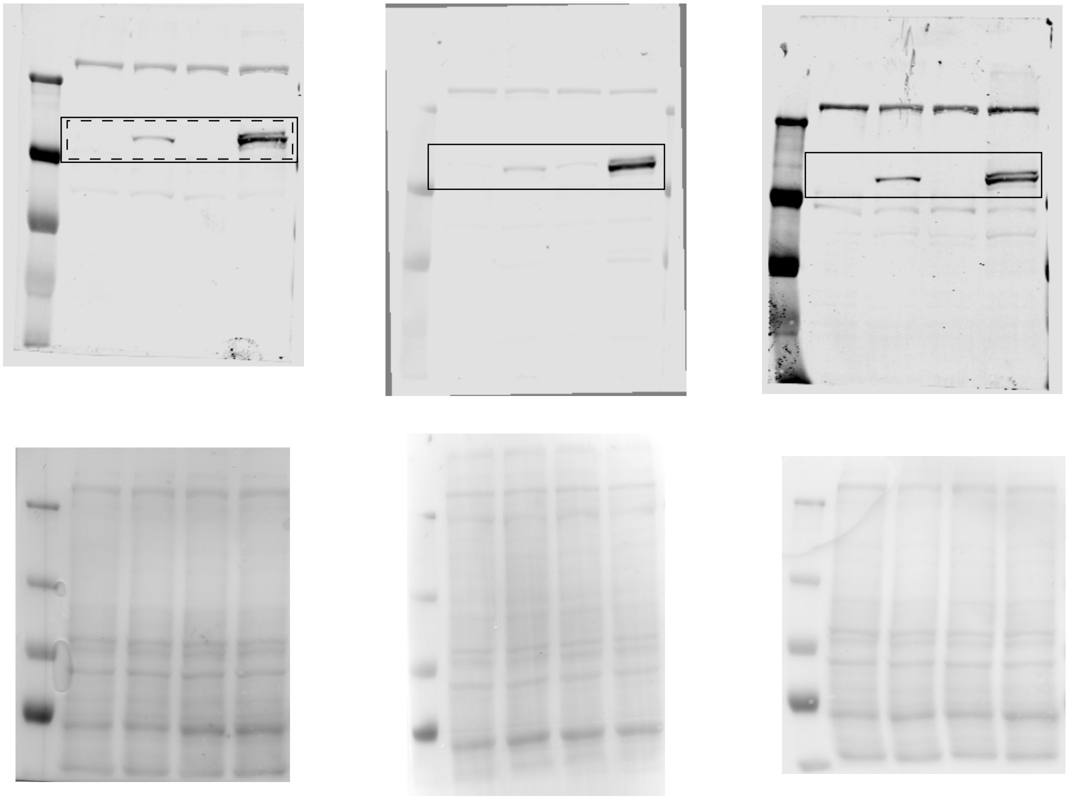

used for figure

used for quantification

Figure 4 - figure supplement 3 Western blots
