# Supplementary material for: Global cellular response to chemical perturbation of PLK4 activity and abnormal centrosome number
Source: eLife. 2022 Jun 27;11:e73944. doi: 10.7554/eLife.73944 (PMC9236612; doi:10.7554/eLife.73944)
Supplement: Source data 2. — (.zip file). [file elife-73944-data2.zip › TkachJM_source_Western/Figure7_supp_western.pdf]

1A

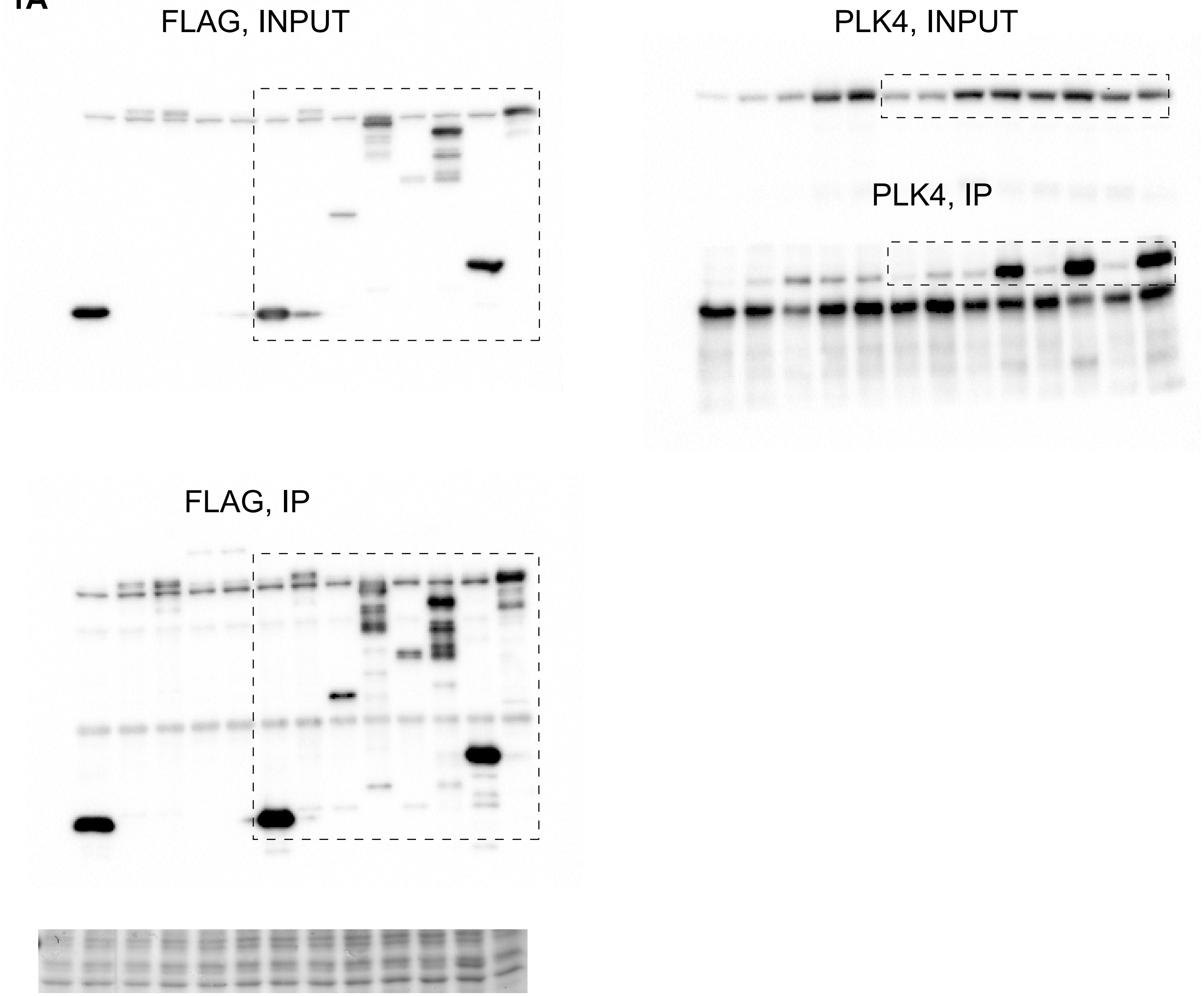

Figure 7 - figure supplement 1 Western blots

1F

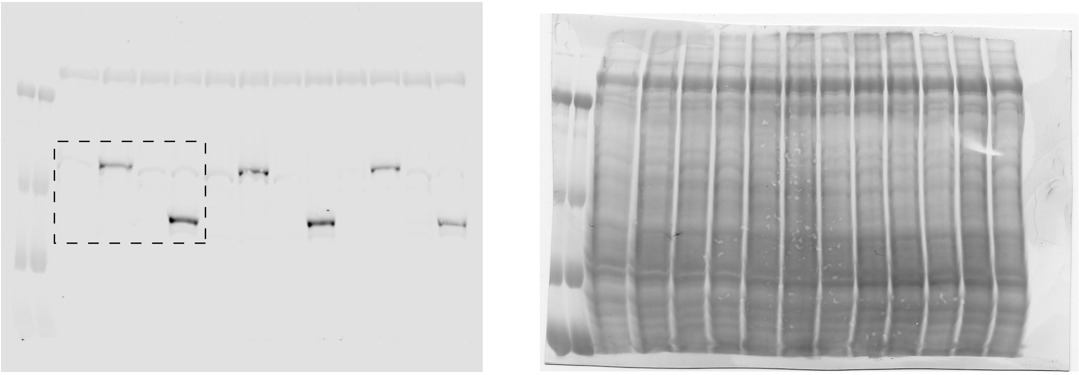

Figure 7 - figure supplement 1 Western blots
